# Phenotypic and Genome-Wide Analysis of an Antibiotic-Resistant Small Colony Variant (SCV) of *Pseudomonas aeruginosa*

**DOI:** 10.1371/journal.pone.0029276

**Published:** 2011-12-15

**Authors:** Qing Wei, Saeed Tarighi, Andreas Dötsch, Susanne Häussler, Mathias Müsken, Victoria J. Wright, Miguel Cámara, Paul Williams, Steven Haenen, Bart Boerjan, Annelies Bogaerts, Evy Vierstraete, Peter Verleyen, Liliane Schoofs, Ronnie Willaert, Valérie N. De Groote, Jan Michiels, Ken Vercammen, Aurélie Crabbé, Pierre Cornelis

**Affiliations:** 1 Research Group Microbiology, VIB Department of Structural Biology, Department of Bioengineering Sciences, Vrije Universiteit Brussel, Brussels, Belgium; 2 Chronic Pseudomonas Infections, Helmholtz Centre for Infection Research, Braunschweig, Germany; 3 Twincore, Center for Experimental and Clinical Infection Research, Helmholtz Center for Infection Research and the Medical School Hannover, Hannover, Germany; 4 School of Molecular Medical Sciences, Centre for Biomolecular Sciences, University of Nottingham, Nottingham, United Kingdom; 5 Functional Genomics and Proteomics, Faculty of Sciences, K.U. Leuven, Leuven, Belgium; 6 Structural Biology Brussels, VIB Department of Structural Biology, Vrije Universiteit Brussel, Brussels, Belgium; 7 Centre of Microbial and Plant Genetics, K.U. Leuven, Heverlee, Belgium; 8 The Biodesign Institute, Center for Infectious Diseases and Vaccinology, Arizona State University, Tempe, Arizona, United States of America; Institute for Genome Sciences, University of Maryland School of Medicine, United States of America

## Abstract

**Background:**

Small colony variants (SCVs) are slow-growing bacteria, which often show increased resistance to antibiotics and cause latent or recurrent infections. It is therefore important to understand the mechanisms at the basis of this phenotypic switch.

**Methodology/Principal Findings:**

One SCV (termed PAO-SCV) was isolated, showing high resistance to gentamicin and to the cephalosporine cefotaxime. PAO-SCV was prone to reversion as evidenced by emergence of large colonies with a frequency of 10^−5^ on media without antibiotics while it was stably maintained in presence of gentamicin. PAO-SCV showed a delayed growth, defective motility, and strongly reduced levels of the quorum sensing *Pseudomonas* quinolone signal (PQS). Whole genome expression analysis further suggested a multi-layered antibiotic resistance mechanism, including simultaneous over-expression of two drug efflux pumps (MexAB-OprM, MexXY-OprM), the LPS modification operon *arnBCADTEF*, and the PhoP-PhoQ two-component system. Conversely, the genes for the synthesis of PQS were strongly down-regulated in PAO-SCV. Finally, genomic analysis revealed the presence of mutations in *phoP* and *phoQ* genes as well as in the *mexZ* gene encoding a repressor of the *mexXY* and *mexAB*-*oprM* genes. Only one mutation occurred only in REV, at nucleotide 1020 of the *tufA* gene, a paralog of *tufB*, both encoding the elongation factor Tu, causing a change of the rarely used aspartic acid codon GAU to the more common GAC, possibly causing an increase of *tufA* mRNA translation. High expression of *phoP* and *phoQ* was confirmed for the SCV variant while the revertant showed expression levels reduced to wild-type levels.

**Conclusions:**

By combining data coming from phenotypic, gene expression and proteome analysis, we could demonstrate that resistance to aminoglycosides in one SCV mutant is multifactorial including overexpression of efflux mechanisms, LPS modification and is accompanied by a drastic down-regulation of the Pseudomonas quinolone signal quorum sensing system.

## Introduction

*Pseudomonas aeruginosa* is a ubiquitous Gram-negative bacterium found in diverse ecological habitats such as soils, marshes and coastal marine waters. As an opportunistic pathogen, *P. aeruginosa* is able to infect humans, animals and plants [1], [2], [3]. *P. aeruginosa* is a primary nosocomial diseases causative agent and represents the major cause of morbidity and mortality in patients with cystic fibrosis (CF). *P. aeruginosa* produces a large panel of secreted virulence factors like the phenazine pyocyanin, the siderophore pyoverdine, elastase, and toxins. It is also characterized by its high level of drug resistance involving the formation of antibiotic-resistant biofilms resulting from the emergence of phenotypic variants [2], [3]. During the course of infection, *P. aeruginosa* can efficiently adopt diverse strategies to evade antimicrobial stresses and the host immune system defenses, making it impossible to eradicate this bacterium permanently from CF lungs [2], [4]. Important phenotypic variations can occur during chronic colonization, such as conversion to mucoidy [5], the emergence of persister cells after antibiotics treatment [6], [7] or the occurrence of small colony variants with higher resistance to antibiotics [8], [9], [10], [11], [12]. Compared to wild-type *P. aeruginosa*, SCVs show increased antibiotic resistance, enhanced biofilm formation, reversion to wild-type-like morphotypes, reduced motility, and slow and auto-aggregative growth behavior [13], [14]. SCVs have been isolated from CF lungs or sputum [4], [8], [9], [12], laboratory-grown biofilms [11], [12], [14], *in vitro* selection upon antibiotic exposure [15], [16] or as a consequence of gene inactivation [17], [18]. Clinically, *P. aeruginosa* SCVs have already been proven to associate with chronic infections behaving as persisters in pathogenesis of CF patients and making it almost impossible for clinicians to eradicate the infections [8], [19], [20]. The intracellular second messenger cyclic-di-GMP (c-di-GMP) [21] has been recently shown to be involved in SCV phenotype switching in terms of biofilm formation, reduced motility, and exopolysaccharide (EPS) production [18], [22], [23], [24], [25], [26]. The “phenotypic variant regulator”, PvrR, containing a conserved EAL domain of phosphodiesterase (PDE) involved in the hydrolysis of c-di-GMP, has been identified to control the phenotypic switch from an antibiotic resistant and auto-aggregative rough SCV (RSCV) of *P. aeruginosa* strain PA14 to wild-type-like antibiotics susceptible revertants [15]. Another characteristic driven by the elevated level of c-di-GMP in SCVs is the contribution of two EPS-encoding loci in some *P. aeruginosa* strains (PA2231-PA2245 for *psl* and PA3058-PA3064 for *pel*) to auto-aggregation and hyper adherence phenotypes characterized by increased Congo Red dye binding [27], [28], [29]. Although antibiotics resistance of *P. aeruginosa* has been connected to biofilm formation and linked to phenotypic variation [15], the mechanisms underlying the extremely high antibiotic resistance of SCVs has not been reported extensively due to the unavailability, in some cases, of the WT counterpart for comparison.

In this study, we present the identification of a novel, reversion-prone, *P. aeruginosa* SCV with distinct features, including resistance to various antibiotics, defective motility, and absence of production of the quorum sensing PQS signal molecule. Using a combination of genomic, transcriptomic, proteomic and phenotypic approaches, we provide the first evidence of concerted mechanisms harnessed by this *P. aeruginosa* SCV leading to antibiotic resistance as well as down-regulation of acute virulence genes, probably involving the PhoP PhoQ two component system.

## Results

### Phenotypic characterization of a gentamicin-resistant *P. aeruginosa* PAO1-SCV and large colony pseudo-revertants

**Figure 1:**
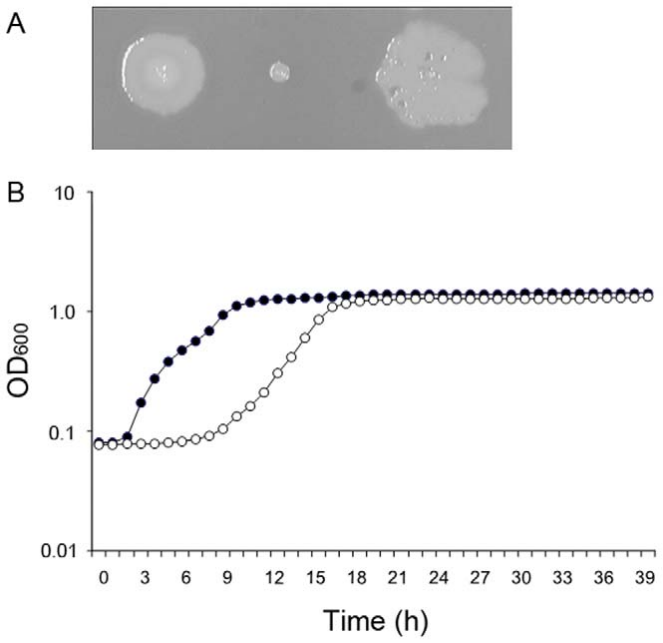
Growth phenotypes of PAO-SCV. A: Comparison of the sizes of colonies from wild-type (left), PAO-SCV (middle), and a large colony variant originating from PAO-SCV (right). The large colony variant shows evidence of autolysis. B: Growth of wild-type (•) and PAO-SCV (○) in LB liquid medium measured in the Bioscreen.

Following sub-culturing *P. aeruginosa* PAO1 (ATCC 15692) in the presence of high-concentration of gentamicin (200 µg ml^−1^, Gm), we isolated a Gm-resistant SCV designated PAO-SCV, which formed small (ca. 1/5 of the wild-type diameter), smooth colonies after three days of incubation at 37°C on LB agar plates (Figure 1A). PAO-SCV grown in liquid LB also showed a delayed entry in exponential phase compared to the wild-type (Figure 1B).

**Figure 2:**
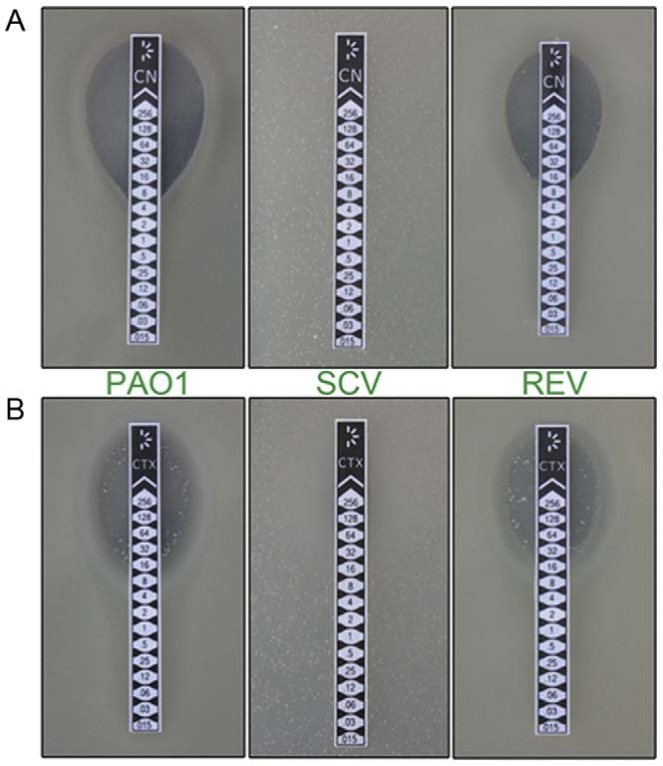
Sensitivity to antibiotics of wild-type, PAO-SCV, and PAO-REV. Sensitivity to gentamicin (A) and cefotaxime (B) of wild type PAO1 (left), PAO-SCV (middle), and one pseudo-revertant (right). The pseudo-revertant and wild-type are sensitive to both antibiotics while PAO-SCV is resistant. Notice the presence of large colonies in the bacterial lawns corresponding to PAO-SCV.

PAO-SCV showed high level of resistance towards gentamicin and cefotaxime (Table 1 and Figure 2). The persistence fraction of PAO-SCV after treatment with the fluoroquinolone antibiotic ofloxacin was approximately 2-fold higher compared to the PAO1 wild-type strain (Figure S1).

**Table 1:** Minimal inhibitory concentrations (MICs) of different antibiotics against wild type (PAO), PAO-SCV and one selected pseudo-revertant (REV).

Antibiotic	PAO	PAO-SCV	REV
Piperacillin	8	8	8
Cefotaxime	16	32	16
Ceftazidime	4	4	4
Imipenem	8	8	< = 1
Meropenem	4	2	0.5
Gentamicin	< = 1	>16	8
Ciprofloxacin	<0.25	<0.25	<0.25
Levofloxacin	1	0.5	1

In the absence of Gm large colonies variants tended to appear, characterized by rough contours, at a frequency of 10^−5^ (Figure 1A and Figure 2) on agar plates. The frequency of reversion varied between 1.3×10^−5^ to 8.7×10^−5^ depending on the medium used (LB or CAA) or the incubation temperature (25°C or 37°C). Importantly, no large colonies appeared when the PAO-SCV was grown in the presence of Gm since the cells from large colonies regained Gm and cefotaxime sensitivity (Figure 2). Given its unstable character, PAO-SCV was kept on LB plates supplemented with Gm (200 µg ml^−1^) to avoid the emergence of pseudo-revertants. However, during experiments described below no antibiotic was added (unless mentioned in the text) in order to avoid Gm-induced changes independent of those caused by the SCV phenotype. At the end of experiments cell suspensions were diluted and the number of large colonies counted. When their number was less than 1/10^5^ the experiment was considered to be valid.

### PQS production is strongly decreased in PAO-SCV

**Figure 3:**
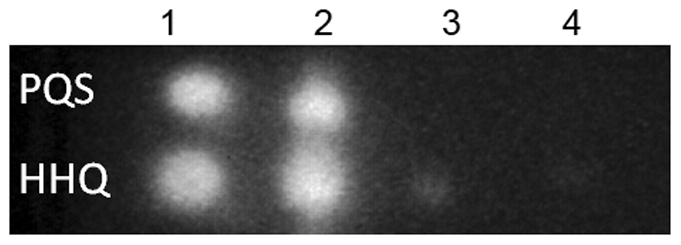
Production of signal molecules. Detection of HHQ and PQS: lane 1: wild-type supernatant from a stationary phase culture in LB, lane 2: same, but from another culture, lane 3 and 4: PAO-SCV supernatant.

We observed that the small colony variant showed reduced production of some known quorum sensing-dependent virulence factors (pyocyanin, pyoverdine, elastase, and a total absence of motility [Figure S2]). Likewise, the PAO-SCV showed strongly reduced virulence using both plants (Belgian endive) and *Drosophila* as hosts (Figure S3). This prompted us to look at the production of quorum sensing signal molecules themselves, including *N*-3-(oxododecanoyl)-L-homoserine lactone (3-oxo-C12-HSL) for the LasR–LasI system and *N*-butyryl-L-homoserine lactone (C4-HSL) for the RhlR–RhlI system [30], [31]. Finally, we also checked the production of 4-quinolones such as 2-heptyl-4-quinolone (HHQ) and 2-heptyl-3-hydroxy-4-quinolone (PQS) [32]. The levels of 3-oxo-C12-HSL and C4-HSL in the cell culture supernatants were similar for the wild-type, PAO-SCV and the pseudo-revertant (results not shown). However, in PAO-SCV a strong decrease in the production of both HHQ and PQS was observed as compared to that of wild-type while the wild type level was restored in the pseudo-revertant (Figure 3).

### Comparison of proteome profiles of PAO-SCV and wild-type *P. aeruginosa*

**Figure 4:**
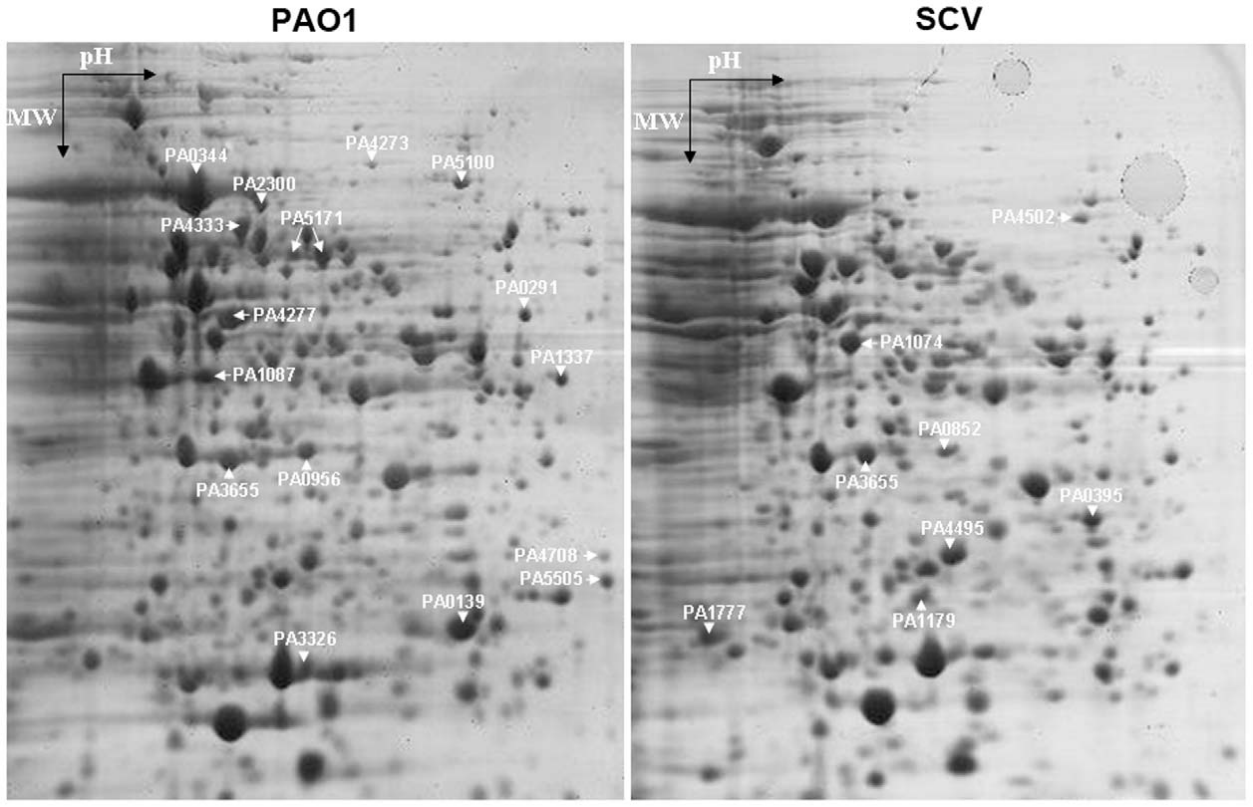
Comparison of proteomes of wild-type and PAO-SCV. 2-D gel electrophoresis of soluble proteins from wild-type and from PAO-SCV grown in LB medium till stationary phase. Spots showing differences are indicated as well as the corresponding PA gene number. See Table 2 for details.

Because profound phenotypic changes were detected in PAO-SCV, we decided to compare the proteomes of PAO-SCV and wild-type cells (Figure 4). After protein identification with MALDI-TOF MS analysis, we found at least 24 differentially expressed proteins, whereby 16 proteins were less abundant and 8 more abundant in PAO-SCV (Table 2). The proteins showing differential abundance are involved in amino acid biosynthesis and metabolism, motility, transport of small molecules and transcriptional regulation. According to this analysis, the two-component response regulator PhoP is one of the most prominently induced proteins in PAO-SCV. Another finding is the over-expression of the major outer membrane protein OprF in PAO-SCV, which is the *P. aeruginosa* major non-specific porin allowing diffusion of various solutes, such as nitrates or nitrites under anaerobic conditions or small oligosaccharides with a molecular weight up to 1519 Da [33], [34]. We also found decreased expression of the anaerobiosis-induced outer membrane porin OprE, which, similarly to OprD, was predicted to be involved in outer membrane permeability of the *β*-lactam antibiotic imipenem and basic amino acids [35], [36]. The SCV proteome showed a drastic decrease in the production of the translation elongation factor Tu, TufB (PA4277) and a less marked decrease in elongation factor Ts (Tsf, PA3655).

**Table 2:** Identification of differentially produced proteins in *P. aeruginosa* PAO-SCV by MALDI-TOF MS peptide mass mapping (PMP).

PA No.	Gene	Protein identification	Matched peptides	Sequence coverage (%)	Total mass (Da)	Localization*	*p*I value
**Down in SCV**							
PA0344		Hypothetical protein	21	47	50,498	Cyt	6.8
PA4273	*rplA*	50S ribosomal protein L1	9	80	93,130	Cyt	8.3
PA5100	*hutU*	Urocanase	34	51	61,554	Cyt	6.0
PA2300	*chiC*	Chitinase	25	53	53,066	Extr	5.2
PA5171	*arcA*	Arginine deiminase	22	52	46,806	Cyt	5.5
PA5171	*arcA*	Arginine deiminase	25	63	46,675	Cyt	5.5
PA4277	*tufB*	Elongation factor Tu	25	68	43,684	Cyt	5.2
PA0291	*oprE*	OprE porin	27	60	49,637	OM	8.7
PA1087	*flgL*	Flagellar hook-associated protein	20	53	47,020	Extr	6.0
PA1337	*ansB*	Glutaminase-asparaginase	22	60	38,620	Per	6.7
PA0956	*proS*	Prolyl-tRNA synthetase	22	37	65,536	Cyt	6.1
PA3655	*tsf*	Elongation factor Ts	27	77	30,691	Cyt	5.2
PA4708	*phuT*	Heme-transport protein	19	68	31,019	Per	6.9
PA5505		Probable TonB-dependent receptor	20	69	28,048	OM	7.8
PA0139	*ahpC*	Alkyl hydroperoxide reductase subunit C	18	66	20,643	Cyt	5.9
PA3326		Endopeptidase Clp chain P	18	70	22,128	Cyt	5.4
**Up in SCV**							
PA4502		Probable binding protein component of ABC transporter	19	31	58,860	Peri	6.2
PA0852	*cbpD*	Chitin-binding protein CbpD precursor	18	45	42,347	Extr	6.4
PA1074	*braC*	Branched-chain amino acid transport protein BraC	23	70	39,858	Peri	5.6
PA3655	*tsf*	Elongation factor Ts	27	83	30,691	Cyt	5.2
PA0395	*pilT*	Twitching motility protein PilT	16	56	41,644	OM	7.2
PA4495		Hypothetical protein	16	55	24,921	Peri	5.8
PA1179	*phoP*	Two-component response regulator PhoP	18	71	25,748	Cyt	5.3
PA1777	*oprF*	OprF	17	75	23,270	OM	4.8

*Cyt = cytoplasm, Peri = periplasm, IM = inner membrane, OM = outer membrane, Extr = extracellular.

### Genome-wide transcriptional profile of PAO-SCV and PAO1

**Figure 5:**
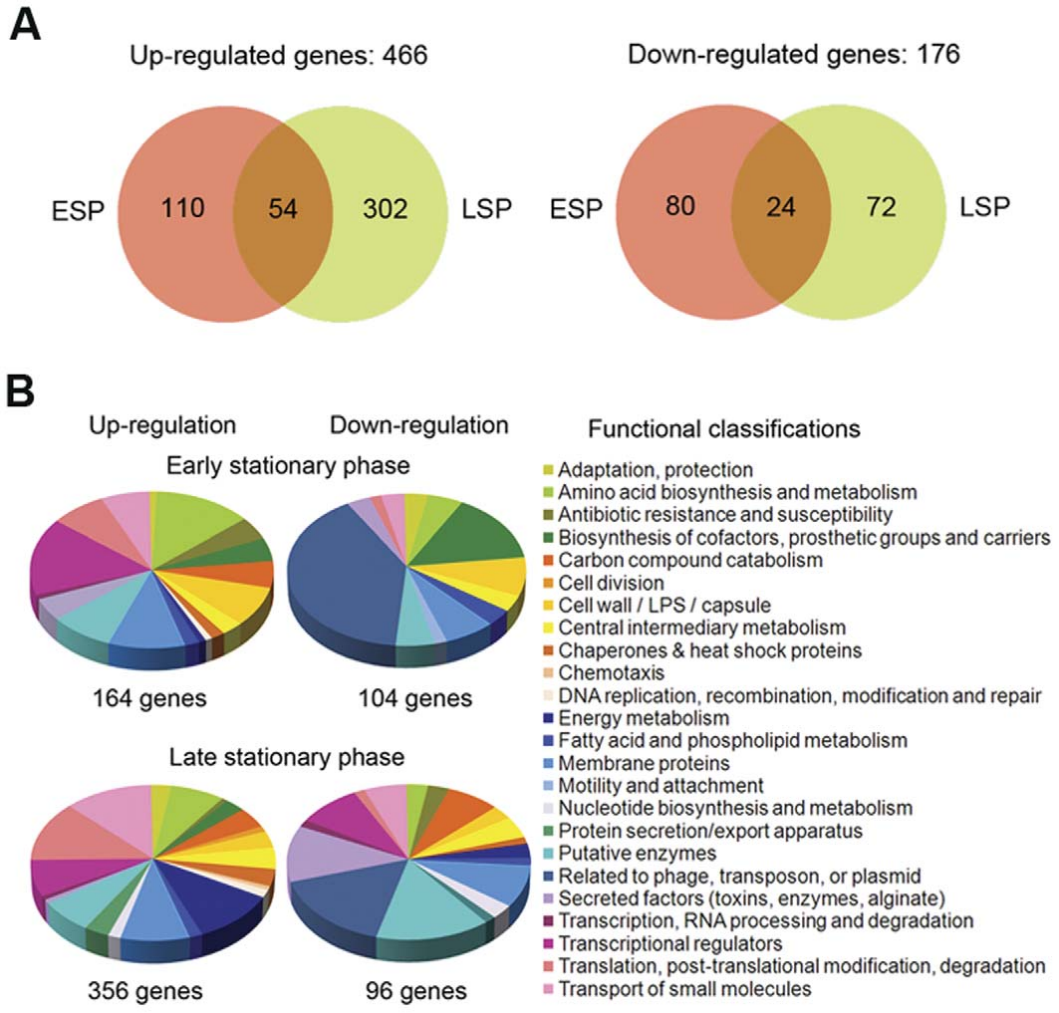
Transcriptome analysis. A: Comparison of up-regulated and down-regulated genes in PAO-SCV in early stationary phase (ESP) and in late stationary phase (LSP). B: Repartition of up- and down-regulated genes in function of genes categories (color coded).

Since some of the differentially produced proteins could already give clue to the changes occurring in the SCV mutant, we decided to further investigate which global changes in gene expression could account for this phenotypic variation. The gene transcription profiles of PAO-SCV and WT strains were compared in early and late stationary-phase of growth, corresponding to incubation times of 20 and 40 h respectively, using *P. aeruginosa* Affymetrix GeneChips. The results are presented using Venn diagrams and pie charts for simplicity, facilitating the understanding and interpretation of the overall genome transcriptional profile [37]. The tables showing the complete lists of differentially expressed genes are shown as supplementary material (Tables S1, S2, S3, S4 and S5). As shown in Figure 5 and in supplementary Tables S1, S2, S3, S4 and S5), during stationary phase, a total of 642 genes representing approximately 12% of the entire genome displayed a differential expression pattern in PAO-SCV compared to that of wild type PAO1 (*P* value <0.05, Student's *t*-test). Among these 642 genes, 466 were up-regulated (≈ 73% of differentially regulated genes, from 2- to 26-fold, see Table S3) and 176 were down-regulated (≈ 27% of differentially regulated genes, from 2- to 16-fold, see Table S4). Interestingly, remarkable differences were observed for up-regulated genes (Figure 5B), among which 356 genes were found to be highly expressed during late stationary phase while only 164 genes were up-regulated during early stationary phase as compared to the wild-type. Genes involved in amino acid biosynthesis and metabolism showed an increased transcription level in both early and late stationary phase of growth of PAO-SCV (Table S2). Genes involved in antibiotic resistance and genes coding for membrane proteins were highly expressed in the SCV mutant in early stationary phase. Conversely, some genes involved in the production of secreted factors and those related to phage, transposon and plasmids were expressed at a lower level in PAO-SCV compared to the wild-type.

**Figure 6:**
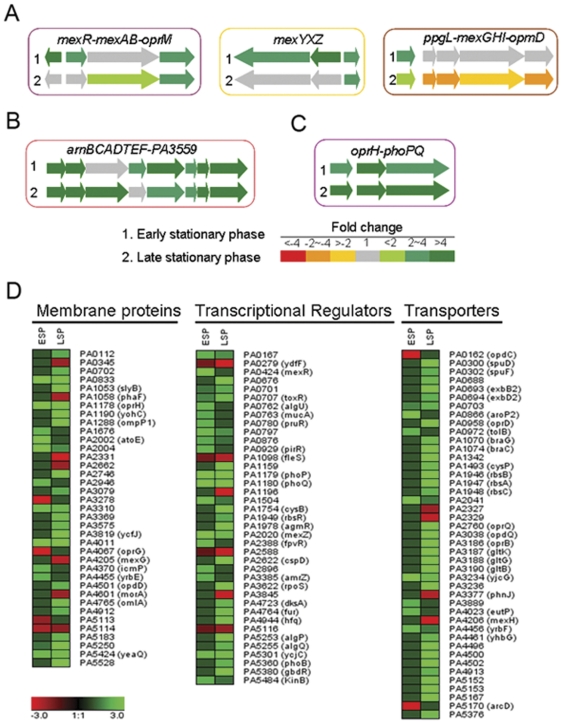
Analysis of differentially expressed genes involved in transport, efflux, and membrane modification. A: Differentially expressed genes corresponding to efflux systems. The *mexXY* and *mexAB*-*oprM* genes are up-regulated while the *mexGHI*-*opmD* genes are down-regulated in PAO-SCV. B: Up-regulation of the *arnBCADTEF*-PA3559 operon for lipid A modification in PAO-SCV. C: Up-regulation of *oprH*-*phoPQ* operon in PAO-SCV. D: Hierarchical clustering of differentially-expressed genes in PAO-SCV corresponding to membrane proteins, transcriptional regulators, and transporters.

Figure 6A shows that some of the genes known to be involved in antibiotic resistance are up-regulated in the PAO-SCV. These could be classified into four different functional groups, linked to four distinct resistance mechanisms (See lists of selected genes in Table S3). Among these are efflux pump systems genes known to contribute to resistance to aminoglycosides, including *mexAB-oprM* and *mexXY* and their respective *mexR* and *mexZ* regulatory genes [38]. The observed higher expression of these efflux pumps is in agreement with the results showing a higher resistance to all aminoglycosides and to the cephalosporin antibiotic cefotaxime (Figure 2 and Table 1). We also found that the *cat* gene encoding the chloramphenicol acetyl transferase is up-regulated in PAO-SCV, both in exponential and stationary phase. Interestingly, expression of another resistance-nodulation-cell division (RND) efflux pump, MexGHI-OpmD, is reduced in PAO-SCV in late stationary phase. This efflux system has been shown to be important for PQS-mediated signaling, pyocyanin production, and is thought to be a general phenazine transporter, including pyocyanin [39], [40], [41]. Again, this observation is in line with the reduced production of pyocyanin by PAO-SCV and the quasi-absence of HHQ and PQS in culture supernatants (Figure 3). Among PAO-SCV up-regulated genes are those involved in LPS modification, including *migA* (PA0705) encoding a glycosyl transferase, and the gene cluster PA3552-PA3559 (*arnBCADTEF*-PA3559, Figure 6B), which are homologues of the *pmrHFIJLKM* genes of *Salmonella enterica* involved in lipid A modification [42], [43], [44]. Interestingly, *phoP*-*phoQ*, together with the upstream porin protein gene *oprH* was markedly up-regulated throughout the stationary phase in PAO-SCV, forming the third functional group, and explaining the overexpression of *migA* and *arnBCADTEF*-PA3559 (Figure 6C). As already mentioned, higher levels of the transcriptional regulator PhoP were also detected by 2D-PAGE analysis. The PhoP-PhoQ system is known to be involved in aminoglycoside resistance in *P. aeruginosa* [45].

A fourth functional group of genes markedly up-regulated in PAO-SCV included those encoding membrane proteins, transcriptional regulators and transporters of small molecules (Figure 6D). More specifically, several genes encoding outer membrane proteins are up-regulated in PAO-SCV: the previously mentioned *oprH*, *oprD*, PA1198 (encoding a lipoprotein), *oprQ*, *opdQ*, *opdP*, and the lipoprotein gene *omlA*. OprQ, and OpdP belong to the OprD family and have been proposed to contribute to the transport of arginine [46]. In this context, it is interesting to note that the genes PA5152 (ABC transporter, ATP binding component), and, to a large extent, PA5153 (periplasmic binding protein), probably involved in the transport of arginine, are also up-regulated.

**Figure 7:**
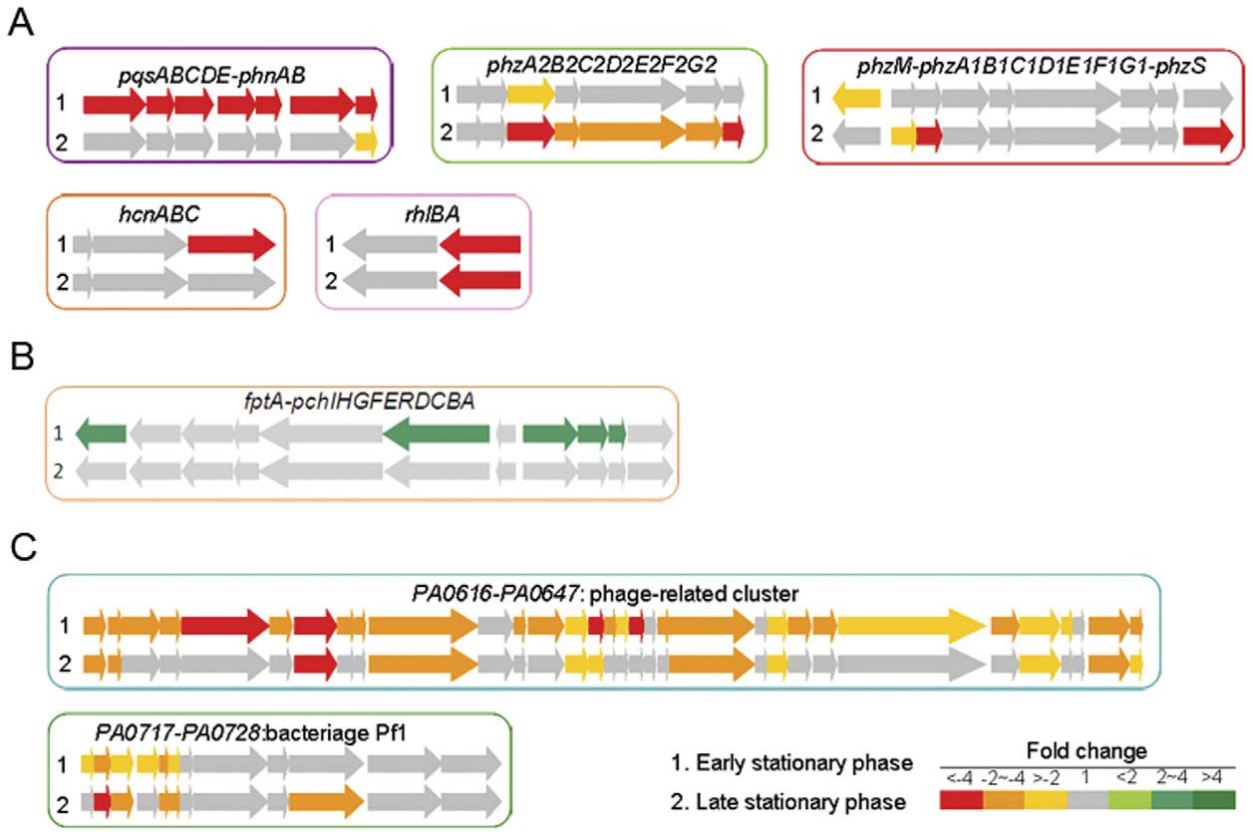
Analysis of down-regulated genes in PAO-SCV. A: Down-regulation of quorum-sensing-regulated genes in PAO-SCV: *pqsABCDE*-*phnAB* for the biosynthesis of PQS, the two phenazine biosynthesis operons (*phz*), the rhamnolipid production *rhlA* gene, and the hydrogen cyanide production gene (*hcn*). B: Up-regulation of pyochelin siderophore biosynthesis (*pch*) and uptake (*fptA*) genes in PAO-SCV. C: Down-regulation of two phage-related clusters of genes in PAO-SCV.

The transcriptome analysis not only provided insights into the PAO-SCV mechanisms involved in aminoglycoside-resistance, but also explained some of the prominent phenotypic changes. As shown in Figure 7A, transcript levels of the *pqsABCDE* genes as well as for the two neighboring anthranilate synthase genes *phnA* and *phnB* were strongly reduced in PAO-SCV, in line with the results presented in Figure 3 showing a strong decrease in HHQ and PQS production. As a result of the down-regulation of PQS genes (*pqsA-E*, *pqsH*, *phnAB*), genes such as *lasA* (coding for elastase), *phzC*2-*G*2, *phzB*1, *phzS* (for pyocyanin biosynthesis), *hcnC* (for HCN production) and *rhlA* (for rhamnolipids synthesis) were also down-regulated. Lower rhamnolipid production could also partly explain the observed decreased swarming motility and the absence of channels in PAO-SCV biofilms [47], [48]. In agreement with the absence of changes in AHLs production, the transcription of *lasI* and *rhlI* coding for the 3-oxo-C12-HSL and C4-HSL synthases was unchanged.

Some genes involved in energy generation via respiration were also differentially regulated since we observed a higher expression in PAO-SCV of cytochromes genes such as PA0105-0108 (encoding cytochrome *c* oxidase subunits I–III), PA1175-1177 (*napDFE* encoding the components of nitrate reduction), and a marked up-regulation of PA1983 *(exaB*, encoding a cytochrome *c*550). Other genes involved in energy generation were found down-regulated in PAO-SCV such as PA4133 (*ccoN*).

Another interesting finding is the differential expression of genes associated with biofilm formation. We found that in PAO-SCV, flagellar synthesis genes expression was reduced compared to wild-type PAO1 which was also confirmed by proteomic analysis (see Figure 5 and Table 2). This result could explain the total absence of motility of PAO-SCV (Figure S2). The third interesting functional group is formed by phage-related genes including phage those involved in Pf1 phage production and the PA0616-PA0647 cluster, the expression of which was greatly reduced in PAO-SCV compared to wild-type (Figure 7C).

### Validation of microarray results via Quantitative RT PCR

**Figure 8:**
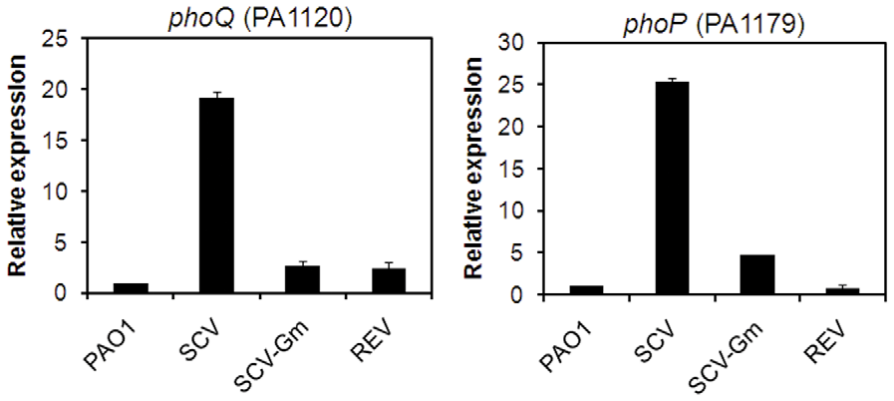
Analysis of *phoP* and *phoQ* gene expression. Quantitative real time PCR analysis of *phoP* and *phoQ* gene expression in wild type PAO1, in PAO-SCV, PAO-SCV grown in the presence of Gm (20 µg ml).

Quantitative real time PCR was used to measure the level of transcripts of the *phoP* and *phoQ* genes in wild type, SCV, and one pseudo-revertant. As shown in Figure 8, the level of *phoP* and *phoQ* transcription was increased in the SCV while the levels were similar for wild-type and the revertant large colony variant.

### Whole genome analysis of *P. aeruginosa* SCV

The genome of the originally selected pseudo-revertant (REV) of PAO-SCV was fully sequenced using the Illumina Genome Analyzer. The choice to re-sequence the revertant only was justified by the fact that it should contain all mutations present in three strains (PAO1, PAO-SCV, revertant), and indels and single nucleotide polymorphisms (SNPs) could be easily checked for their presence in the genomes of PAO-SCV and its clonal wild-type using a combination of PCR amplification and Sanger sequencing. A limited list of sequence variations in relation to the PAO1 sequence was found (Table 3), most of which were already detected when we re-sequenced these regions in our own PAO1 lab strain and in the PAO1 strain of Chronic *Pseudomonas* Infection Group in Helmholtz Infection Research Centre [49]. In order to exclude the possibility that genetic variations have been overlooked due to the limitations of reference based read mapping, the reads were additionally assembled in a *de novo* approach using Velvet and the resulting contigs were aligned with the PAO1 genome to find genetic variations. Finally, eight changes remained that were unique to REV after elimination of the mutations also found in PAO1 wild-type (Table 3). For seven of these regions, PCR amplification and re-sequencing via the Sanger method revealed that these differences in sequence were present in both REV and SCV. In only one instance, a mutation was found that was unique to REV, causing a base change at position 1020 of the *tufA* gene (PA4277), a paralog of *tufB* (PA4265). The two genes are nearly identical in sequence, except at this position where *tufB* has a GAC codon for aspartate while tufa has the rarely used codon GAT at the same place. In REV, a mutation caused a silent mutation in *tufA*, changing the GAT codon to GAC, like in the *tufB* gene. However, no additional variations were identified and all contigs aligned to at least one genomic region of PAO1 excluding the possibility of an unrecognized insertion of exogenous genetic elements (e.g., phages or transposons). The detection of genetic variations usually assumes a clonal population and SNPs and indels of low abundance are mostly interpreted as sequencing errors and excluded from the analysis. Nevertheless, the absence of REV specific mutations (except in one case) may be due to several mutations that independently cause the phenotypic conversion and are present in different clones coexisting in a mixed population. The original data were therefore re-analyzed to identify SNPs and indels that were present in at least 10% of the reads. However, this analysis also identified only those variations that were already detected in the initial approach.

**Table 3:** List of genetic variations in *P. aeruginosa* SCV revertant.

Locus^a^	Gene name	Product	Position^b^	Nucleotide	Ref. base^c^	variant base	Type	Protein effect^d^	Position [AA]^e^	SCV^f^	WT
PA0748	*mmsR*	Still frameshift probable transcriptional regulator	816532	425	G	C	Single insertion	Frameshift	142	+	+
PA1179	*phoP*	Two-component response regulator PhoP	1277728	41	A	G	SNP	H->R exchange	14	+	-
PA1180	*phoQ*	Two-component sensor PhoQ	1279044	683			39 bp deletion	In frame deletion	228	+	-
PA1385		Probable glycosyl transferase	1505156	552	C	T	SNP	Silent mutation	184	+	ND
PA2020	*mexZ*	Probable transcriptional regulator	2213076	400	C	T	SNP	Nonsense mutation	134	+	-
PA2046-PA2047		Intergenic region	2239547	280	T	G	SNP			+	ND
PA2141		Hypothetical protein	2356684	517	C	-	Single deletion	Frameshift	173	+	+
PA4265	*tufA*	Elongation factor Tu	4767985	1020	T	C	SNP	Silent mutation	340	-	-

a Gene locus, gene name and product description are extracted from Pseudomonas Genome Database (http://www.pseudomonas.com).

b Chromosomal position (nt).

c Ref. base, reference base.

d Change of protein sequence.

e Position relative to first amino acid of the protein.

f Found in SCV as well after Sanger sequencing.

ND, not determined.

Interestingly, changes were identified in *phoPQ* and *mexZ*, in line with the results of the transcriptional analysis. Specifically, the *phoP* gene contains a SNP which confers a histidine (H) to arginine (R) change while, remarkably, *phoQ* harbors an in-frame 39-bp deletion in its coding sequence, deleting a 13 amino acids sequence RLLRSEHKQRERY between residues 226 and 239. In PAO-SCV the *mexZ* gene was inactivated by the introduction of a stop codon, which could explain the over-expression of the MexXY pump involved in aminoglycosides and fluoroquinolone resistance.

## Discussion

The SCV phenotype observed in this study is reminiscent of the observations made by Tarighi *et al*. who found that knocking out the the *ppgL* gene (PA4204) of *P. aeruginosa* caused a SCV phenotype with the apparition of large colony variants [17]. In this particular case the SCV phenotype was thought to be due to the accumulation of a toxic intermediate: gluconolactone [17]. This observation suggests that SCV phenotypes can be the results of exposures to different stresses. In *P. aeruginosa*, several mechanisms of aminoglycoside resistance have been described: resistance through efflux systems, by alteration of porins or outer membrane properties (including LPS modification), resistance through chromosomal mutations of regulatory genes, and resistance through enzymatic drug modification, including both intrinsic and acquired resistance [50], [51], [52], [53], [54], [55]. *P. aeruginosa* can use these mechanisms in combination, to reach high-level of resistance to certain antibiotics, which is precisely what we observed in this study since we found an overexpression of two efflux systems (MexXY-OprM, MexAB-OprM, increased expression of the *arnBCADTEF*-PA3559 LPS modification genes, of the porins OprH, OprF, and decreased expression of OprE. The tripartite efflux pump MexXY-OprM is known to be a major contributor to aminoglycoside resistance in *P. aeruginosa* [56], [57], [58], [59] as well as the efflux of the drug tigecycline [60]. In addition, MexAB-OprM was also proven to confer resistance to β-lactams, fluoroquinolones [51] and to contribute to aminoglycosides resistance [61]. Both efflux pumps share the same efflux porin, OprM [62]. In *P. aeruginosa*, the PhoP-PhoQ two-component regulatory system is known to be induced upon Mg^2+^ starvation to up-regulate the production of the outer-membrane protein OprH and to increase the resistance to the polycationic antibiotic polymyxin B [63]. In addition, PhoP-PhoQ is also involved in resistance to antimicrobial cationic peptides and aminoglycoside antibiotics [45], in good agreement with the higher resistance of PAO-SCV to this class of antibiotics. The *arnBCADTEF*-PA3559 genes could also be involved in conferring a higher resistance to aminoglycosides. Intriguingly, PA3559 encodes a UDP-glucose dehydrogenase that is induced by low concentrations of Mg^2+^ [43] and its expression depends on the PmrA-PmrB two-component regulatory system, which is itself regulated by the PhoP-PhoQ two-component system [64], [65], [66], [67]. The general porin OprF, which is overexpressed in the SCV, has been recently shown to participate in resistance mechanisms to a broad spectrum of antibiotics such as β-lactams, cephalosporins, and fluoroquinolones [68], supporting the role of OprF as an intrinsic antibiotic resistance contributor as well as partially explaining the cefotaxime resistance revealed by phenotypic assays. Interestingly, we observed a down-regulation of the cytochrome *c ccoN* gene (PA4133), which was shown by others to be involved in small colony variant formation leading to higher resistance to aminoglycosides [69]. In PAO-SCV we observed a down-regulation of phage genes compared to the wild-type. The expression of phage genes has been shown to reciprocally associate with biofilm formation and antibiotic resistance as evidenced by Whiteley and colleagues [70]. These authors found that phages genes were up-regulated in mature biofilms as compared to planktonic cultures while they were shown to be down-regulated in biofilms exposed to the aminoglycoside tobramycin. A puzzling observation is the strong down-regulation in PAO-SCV of PQS biosynthesis genes and of the PQS-related efflux pump MexGHI-OpmD [39], which is confirmed by the near absence of PQS production presented in Figure 3. It has been suggested that high production of PQS, which results in an autolysis phenotype, could be explained by the induction of prophages [22], which fits with the results presented here.

Collectively, our whole genome expression analysis of PAO-SCV versus wild-type PAO1 allowed us to get a good correlation between phenotypic traits (antibiotic resistance, PQS and virulence factors production), proteomic and gene expression data. Intriguingly, we found only one mutation to be present in the pseudo-revertant only. This mutation could cause a higher translation rate of the *tufA* messenger RNA since it changed a rarely used GAU codon for aspartate to the more frequently used GAC. It is also interesting to notice that in the SCV proteome there was a strong decrease of the TufB protein (Figure 4). In *Salmonella enterica*, the translation factor Ef-Tu is also encoded by two paralogous genes, *tufA* and *tufB*, and it has been shown that mutations in *tufB*, which impair growth, can be rescued by compensatory mutations in the RNAse E gene or in *tufA* [71], situation similar to what we observed here. However, we have to be cautious before concluding that the *tufA* mutation is the only cause of the reversion associated with a higher growth rate. Our results do not explain either why the level of the TufB protein is strongly decreased in the SCV since no changes were found at the transcriptional level. We should not exclude either the possibility of a phenotypic switch without mutation, like the recently described bi-stable phenotypic switch due to the LysR regulator BexR [72]. Phenotypic switches have been described in *Pseudomonas brassicacearum* leading to two types of colonies with different abilities to colonize plant roots [73]. In rhizosphere pseudomonads phase variation is commonly observed and in one case the involvement of GacS/GacA two-component system as well as MutS and RpoS in phase variation has been described [74], [75], [76]. However, we could not detect any nucleotide change in *gacA*/*gacS* or in *mutS* and *rpoS*, which suggests that the switch has another, unidentified, origin and excluding the possibility that the SCV strain has a hypermutator phenotype.

In conclusion, our study demonstrates that the SCV phenotype could arise due to the accumulation of several mutations and that the resistance to aminoglycosides expressed by the SCV clone is multifactorial.

## Methods

### Bacterial strains and culture conditions

*P*. *aeruginosa* PAO1 Strain (ATTC 15692) and its gentamicin-resistant mutant PAO-SCV were used in this study. *P*. *aeruginosa* strains were grown at 37°C in Luria-Bertani (LB) broth or on LB agar plates, iron poor casamino acids (CAA) medium (Difco Laboratories) or *Pseudomonas* agar medium (Difco Laboratories). The antibiotics gentamicin (Gm) at 200 µg ml^−1^ and spectinomycin (Sp) at 50 µg ml^−1^ were used when necessary. Growth rate of three replicates for each strain was monitored spectrophotometrically (Bioscreen C, Thermo Labsystems).

### Motility assay

Swarming, swimming and twitching motility were determined as previously described [77]. To investigate swarming motility, 4 µl of overnight cultures of *P. aeruginosa* grown in LB (1×10^9^ cells) were placed in the center of 0.4% agar LB or CAA plates while swimming motility was evaluated using 0.3% agar LB or CAA plates. For twitching motility, LB or CAA plates containing 1.5% agar were inoculated with a toothpick by stabbing the plates. The plates were then incubated at 37°C. In the case of twitching, after incubation the LB- or CAA-agar media was removed from the plates and plates were stained with 1% crystal violet (Merck) in 33% acetic acid for a minimum of 20 min. Spreading of bacteria from the inoculation point was measured and pictures were taken. Three independent experiments were performed.

### Detection and analysis of signal molecules

Rapid detection of *N*-acyl homoserine lactones (AHLs) in filter sterilized (0.2 µm pore-size filters) culture supernatants was done using AHL reporter plate bioassays by either *E. coli* JM109 carrying the plasmid pSB401 for the detection of *N*-(butanoyl)-L-homoserine lactone (C4-HSL) [78] or *E. coli* MH155 [79] for the detection of *N*-(3-oxododecanoyl)-L-homoserine lactone (3-oxo-C12-HSL). For accurate AHL quantification, 100 ml of acidified filter-sterilized culture supernatants were extracted with equal volumes of dichloromethane. The organic phase was removed and dried by evaporation in vacuum. Extracts were re-dissolved in 1 ml of 50% acetonitrile. Thin-layer chromatography (TLC) plates Silica gel 60 F_254_ (Merck) and RP-18 F_245_ (Merck) were used for detection of 3-oxo-C12-HSL and C4-HSL, respectively. Twenty µl of extracted AHLs were fractionated on TLC plates. After development in a solvent mixture of methanol/water (60∶40, vol/vol), the plates were dried and overlaid with 50 ml of soft top LB-agar mixed with 1 ml of overnight culture of an *E. coli* MH155 strain harboring the reporter plasmid pUCP22*Not*I-P*_lasB_*::*gfp*(ASV)P*_lac_* ::*lasR* (to detect 3-oxo-C12-HSL) or 1 ml of overnight culture of *E. coli* JM109 pSB401 (to detect C4 and C6-HSL). After 18 h incubation at 37°C the production of 3-oxo-C12-HSL was detected under UV by visualization of green fluorescent spots and production of C4 and C6 was visible by the production of light.

The alkyl-hydroxy quinolones PQS and HHQ were extracted from 10 ml of early stationary phase filtered supernatants by adding equal volumes of acidified ethyl acetate. The organic phase was dried and the residue re-suspended in 50 ml methanol. Ten µl samples of this extract were spotted onto normal phase silica 60 F_254_ (Merck) TLC plates, pre-treated by soaking in 5% K_2_HPO_4_ for 30 min and activated at 100°C for 1 h. Extracts were separated using a dichloromethane:methanol (95∶5, vol/vol) solvent system until the solvent front reached the top of the plate. PQS was visualised under UV light and specific detection was done using soft top LB-agar including 1 ml of overnight culture of a *P. aeruginosa lecA*::*lux ΔpqsA* strain as bioreporter [80]. Bioluminescence was detected and quantified with a Bio Imaging System (Syngene). Amounts of C4-HSL, C6-HSL, 3-oxo-C12-HSL and PQS were determined by measuring the diameter of the spots.

### Persistence assay

The persistence assay was performed essentially as described previously [6]. Shortly, cultures were grown overnight at 37°C in 100 ml LB medium in Erlenmeyer flasks. One mL of a stationary phase culture was treated with 10 µL of ofloxacin at a final concentration of 5 µg mL^−1^; a control treatment was performed with sterile water. Both treatments were performed at 37°C, shaking at 200 rpm, during five hours, after which the number of colony forming units were determined by plate counts. The persister fraction is defined as the number of surviving cells after treatment with ofloxacin, divided by the number of cells after the control treatment. The relative persister fraction for each strain is the persister fraction of the strain divided by that of the wild-type. The mean relative persister fraction is calculated as the inverse logarithm of the mean of the logarithmic values of these relative persister fractions of separate experiments. The mean relative persister fractions are displayed with the bars representing the 25^th^ and 75^th^ percentiles as shown in Figure S1. Each experiment was repeated at least three times.

### Proteome analysis by two-dimensional (2D) gel electrophoresis

*P. aeruginosa* cells were harvested in early stationary phase by centrifugation (4,000 *g*, 10 min, 4°C) and washed three times with Tris-HCl buffer (pH 8.0). To prepare extracts of cellular proteins, bacterial cells were washed twice in PBS buffer (pH 8.0) and re-suspended in a solution containing 40 ml of 2.5 mM Tris-HCl (pH 8.0), one tablet of protease inhibitor (Sigma), 80 µl of 0.5 M Na_2_EDTA, and 400 µl of DNase at 10 mg ml^−1^. After lysis of the cells by sonication with a Branson Sonifier 250, each suspension was centrifuged (2,500 *g*, 15 min, 4°C) to remove the cell debris and unbroken cells. The supernatant was then subjected to a second centrifugation (50,000 *g*, 40 min, 4°C) to remove the insoluble components, and the protein concentration in the resulting supernatant was determined by a Bradford Protein assay (Bio-Rad). Deoxycholic acid (sodium salt) was added to a final concentration of 0.2 mg ml^−1^. After 30 min of incubation on ice, the proteins were precipitated by addition of 6% (wt/vol) trichloroacetic acid and incubated at 4°C for 2 hr. After centrifugation (18,000 *g,* 30 min, 4°C) the precipitated proteins were re-suspended in distilled water, and eight volumes of cold acetone (−20°C) were added. After incubation at −20°C for 2 h, the mixture was centrifuged (3,500 *g,* 20 min, 4°C), and the pellet was allowed to dry for 5 min before it was dissolved in an appropriate amount of solubilisation buffer. After centrifugation (50,000 *g,* 40 min, 4°C) to remove the insoluble components, the protein concentration of the remaining supernatant was determined. Protein extracts were either used immediately for 2-D gel electrophoresis or stored at −80°C. Isoelectric focusing was performed with the IPGphor system and Immobiline DryStrip gel strips (GE Healthcare). Equal quantities of solubilised proteins from the different *P. aeruginosa* strains were diluted to obtain a final volume of 360 µl with solubilisation solution and applied to the Immobiline gel strips by in-gel rehydration. Linear immobilized pH gradients (pH 4 to 7) were used. Thirty to 50 µg of protein was applied for analytical gels (silver staining), and 200 to 500 µg of protein was loaded for Coomassie staining. After rehydration under silicone oil for 10 hr, the proteins were focused for a total of 120 kV/h at 20°C. The proteins were reduced by equilibration of the strips in equilibration solution (6 M urea, 30% glycerol, 2% [wt/vol] sodium dodecyl sulfate, and 1% [wt/vol] dithiothreitol in 0.05 M Tris-HCl (pH 8.8) for 15 min and then carbamidomethylated in the same solution containing 260 mM iodoacetamide for 15 min. The strips were transferred to 12% acrylamide gradient gels and electrophoresis was performed overnight at 125 V at 10°C. Gels were stained with Coomassie brilliant blue solution and spots of interest were then further analyzed through peptide mass fingerprinting according to the described protocol [81]. Peptides examined on a MALDI-TOF mass spectrometer (Bruker) and analyzed by MASCOT (Matrix Science) were used to identify proteins from peptide identifications using the NCBInr database.

### Microarray and quantitative real time PCR analysis

Cultures were grown in triplicate until early (24 h) and late (48 h) stationary phase, respectively, allowing three biological replicates per condition. Total RNA was obtained from the cultures of early and late stationary phase by first treating the cells with RNAprotect Bacteria Reagent (Qiagen) as recommended by the manufacturer. Cells were then lysed and total RNA was extracted using the RNeasy Midi Kit (Qiagen), on-column DNase digestion was performed using the RNase-free DNase Set (Qiagen) according to the manufactures instructions. RNA integrity was assessed using the 2100 bioanalyzer (Agilent Technologies Inc.). cDNA synthesis, fragmentation and labeling were performed according to the supplier protocol for the *P. aeruginosa* Genechip genome array (Affymetrix) at 50°C. Washing and staining of the arrays was performed according to the manufacturer's instructions using a fluidics station 400 (Affymetrix). Slides were scanned using the 2500A GeneArray Scanner (Agilent Technologies Inc.) and Affymetrix MAS 5.0. Data analysis was performed using GeneSpring GX (Agilent Technologies Inc.) in which the scaled data was further normalized by per Chip and per Gene median normalizations. Filtering of genes was performed to find genes that had changed in expression by a magnitude of 2-fold (*P* value<0.05, Student's *t*-test).

Bacterial cells were harvested in stationary phase, bacterial RNA was extracted by using RNeasy Midi Kit (QIAGEN). The purity and concentration of the RNA was determined by spectrophotometry (NanoDrop, Thermo Scientific). First-strand cDNA was reverse transcribed from one microgram of total RNA by using First-strand cDNA Synthesis Kit (Amersham Biosciences, GE Healthcare). qRT-PCR was performed in a Bio-Rad (Hercules, CA, USA) iCycler with Bio-Rad iQ SYBR Green Supermix. For all primer sets, the following cycling parameters were used: 94°C for 3 min followed by 40 cycles of 94°C for 30 s, 55°C for 45 s and 72°C for 30 s, followed by 72°C for 7 min. The outer membrane lipoprotein o*prI* gene was used to normalize gene expression [82] [82] [82] [81]. Amplification products were electrophoresed on 0.8% agarose gels. For statistical analysis of relative gene expression, the 2^−△△*C*T^ method was used [83]. All experiments were carried out in triplicate.

### Whole genome sequencing

Genomic DNA was isolated from thawed pellets using the DNeasy Blood & Tissue Kit (Qiagen) according to the manufacturer's instructions. DNA samples were further prepared and sequenced using paired end sequencing in 76 cycles on a Genome Analyzer II–X (Illumina). Libraries of 300 bp were prepared according the manufacturer's instructions “Preparing Samples for Paired-End-Sequencing”. Cluster generation was performed using the Illumina cluster station, sequencing for read 1 and read 2 on the Genome Analyzer followed a standard protocol. The fluorescent images were processed to sequences using the Genome Analyzer Pipeline Analysis software 1.3.2 (Illumina).

Sequencing the DNA library resulted in a total number of 12,302,376 read pairs of 2×76 nt length. Reads were mapped to the reference genome of strain PAO1, which was obtained from the *Pseudomonas* genome database [84]. The 3′-ends of reads were trimmed using Perl script Trim.pl (http://bioinformatics.ucdavis.edu/index.php/Trim.pl) with the adaptive window option and a quality threshold of 10 to remove sequences of low read quality. Reads pairs containing one or two reads that were trimmed to a length less than 20 nt were discarded leaving 11,991,338 read pairs (97.5%). The free license version of Novoalign (www.novocraft.com) was used for mapping, because this software includes a gapped alignment algorithm, which improves the detection of indels [85]. 11,824,266 read pairs (96.1% of the original reads) were mapped to unique locations resulting in a median read depth (genome coverage) of 237. Single nucleotide polymorphisms (SNPs) and indels were detected using the MAQ software [86] using its built-in functions “cns2snp” and “indelpe”, respectively. Initially, only SNPs with a minimal consensus quality of 30 and indels that were supported by at least 50% of the reads overlapping the indel position were considered for further analysis as potential true positives. All positively filtered SNPs and indels were checked by visual inspection for correct base/indel calling. Genomic regions showing a read depth of less than 30 were also checked by visual inspection for the occurrence of larger indels that cannot be detected by the combination of Novoalign and MAQ alone. Furthermore, the confirmation of positives by Sanger sequencing has been performed and aligned with the sequences extracted from www.pseudomonas.com.

Additionally, the quality-trimmed read pairs were also assembled *de novo* using Velvet [87] yielding 1181 contigs with an n50 of 18.4 kbp and maximum length of 83.0 kbp. SNPs and indels were detected by aligning the contigs to the PAO1 reference genome using mummer3 [88].

### Virulence assays

*P. aeruginosa* strains were grown overnight in LB medium to reach stationary phase at 37°C. After centrifugation, bacterial cells were washed with 10 mM MgSO_4_ and diluted to 10^8^ CFU/ml and then 10 µl of this suspension were injected with a syringe into the main vein of Belgian chicory (*Cichorium intybus*) leaves. The leaves were placed on dishes containing a Whatman filter impregnated with sterilized water. The plates were kept in an incubator at 37°C and watery rot symptoms monitored daily for 72 h.

Virulence was also tested on third instar larvae of the fruitfly (*Drosophila melanogaster*). Larvae were pricked with needles dipped in a concentrated suspension of *P. aeruginosa* strains grown in LB medium with an absorbance of 1.0 at 600 nm. After pricking, the larvae were placed in plates containing a piece of paper impregnated with 10% sugar water. For each condition 20 plates with 10 larvae were used. During 8 h, the number of dead larvae was counted every 30 min. A final count was held 24 h after initial pricking. Data were statistically analyzed by means of repeated measures ANOVA.

GEO Accession Number: GSE34141

## Supporting Information

Figure S1 Mean relative persister fraction of wild-type PAO1 and PAO-SCV after exposure to ofloxacin. See text for details.
(TIF)

Figure S2 Production of virulence factors pyocyanin, pyoverdine, and elastase (A); motility of wild type PAO1 and PAO-SCV on swimming (top), swarming (middle), and twitching (bottom) plates (B); atomic force microscopy images of wild-type and SCV showing the loss of flagella (C).
(TIF)

Figure S3 Virulence of wild-type, PAO-SCV and revertant (A) in plants (*Cychorium intybus*) and (B) of wild-type and PAO-SCV in *Drosophila melanogaster* larvae.
(TIF)

Table S1 Number of differentially expressed genes between *P. aeruginosa* PAO-SCV and its clonal wild-type PAO1 during stationary phase.
(DOC)

Table S2 Functional classification of differentially expressed genes between *P. aeruginosa* PAO-SCV and its clonal wild-type PAO1 during stationary phase.
(DOC)

Table S3 Up-regulated genes in *P. aeruginosa* PAO-SCV compared to its clonal wild-type PAO1 during stationary phase.
(DOC)

Table S4 Down-regulated genes in *P. aeruginosa* PAO-SCV compared to its clonal wild-type PAO1 during stationary phase.
(DOC)

Table S5 Differential expression of selected genes in *P. aeruginosa* PAO-SCV compared to its clonal wild-type PAO1 during stationary phase.
(DOC)
