# Supplementary figures and images for: Phenotypic and Genome-Wide Analysis of an Antibiotic-Resistant Small Colony Variant (SCV) of Pseudomonas aeruginosa
Source: PLoS One. 2011 Dec 15;6(12):e29276. doi: 10.1371/journal.pone.0029276 (PMC3240657; doi:10.1371/journal.pone.0029276)

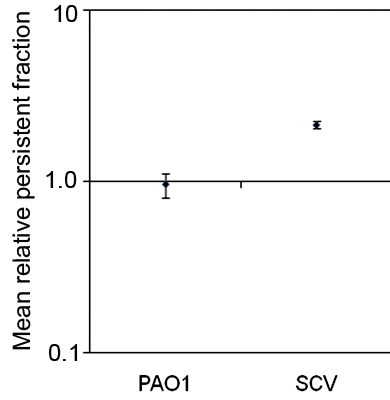

Supplement: Figure S1 — Mean relative persister fraction of wild-type PAO1 and PAO-SCV after exposure to ofloxacin. See text for details. (TIF) [file pone.0029276.s001.tif]

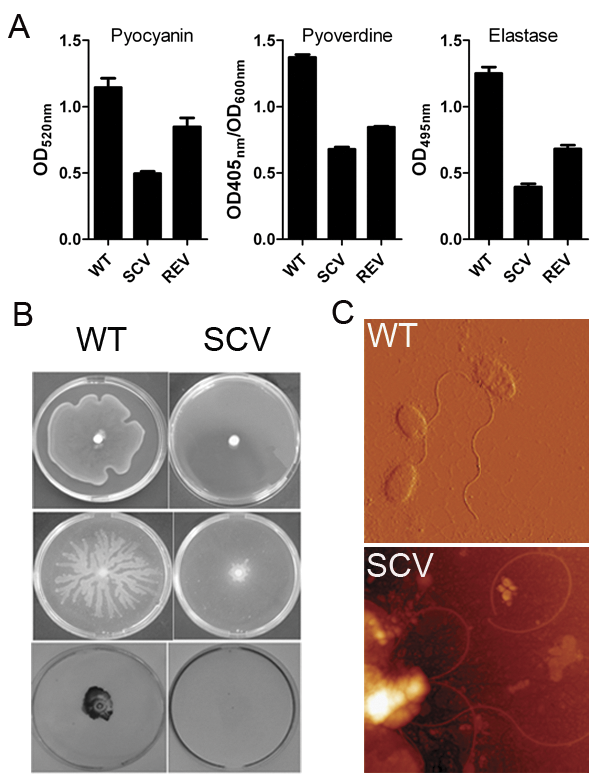

Supplement: Figure S2 — Production of virulence factors pyocyanin, pyoverdine, and elastase (A); motility of wild type PAO1 and PAO-SCV on swimming (top), swarming (middle), and twitching (bottom) plates (B); atomic force microscopy images of wild-type and SCV showing the loss of flagella (C). (TIF) [file pone.0029276.s002.tif]

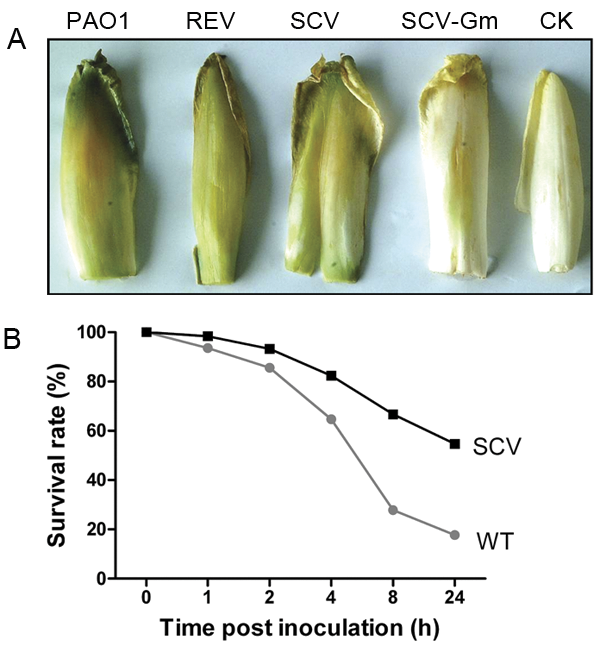

Supplement: Figure S3 — Virulence of wild-type, PAO-SCV and revertant (A) in plants (Cychorium intybus) and (B) of wild-type and PAO-SCV in Drosophila melanogaster larvae. (TIF) [file pone.0029276.s003.tif]
