# Supplementary material for: Phenotypic and Genome-Wide Analysis of an Antibiotic-Resistant Small Colony Variant (SCV) of Pseudomonas aeruginosa
Source: PLoS One. 2011 Dec 15;6(12):e29276. doi: 10.1371/journal.pone.0029276 (PMC3240657; doi:10.1371/journal.pone.0029276)
Supplement: Table S1 — Number of differentially expressed genes between P. aeruginosa PAO-SCV and its clonal wild-type PAO1 during stationary phase. (DOC) [file pone.0029276.s004.doc]

| **Table S1. Number of differentially expressed genes between *P. aeruginosa* PAO-SCV and its clonal wild-type PAO1 during stationary phase** | | | |
| --- | --- | --- | --- |
|  |  |  |  |
|  |  |  |  |
| **Fold change** ^a^ | **Number of expressed genes** ^b^ | | |
|  | **Early SP** | **Late SP** | **SP** |
|  | **SCV/WT** | **SCV/WT** | **SCV/WT** |
| Up-regulated |  |  |  |
| >4 | 28 | 75 | 96 (7 ^c^) |
| 2-4 | 136 | 281 | 384 (33) |
| Total (>2) | 164 | 356 | 466 ^d^ (40+14 ^e^) |
| Down-regulated |  |  |  |
| >4 | 23 | 20 | 41 (2) |
| 2-4 | 81 | 76 | 145 (12) |
| Total (>2) | 104 | 96 | 176 (14+10) |

^a^ Fold change is the arithmetic average of a change in gene expression level that was significant using student T-test (P value less than 0.05).

^b^ SP, stationary phase; SCV: PAO-SCV; WT: wild-type.

^c^ Number in parenthesis indicates the number of overlapping genes between early and late stationary phase with the same fold change level, i.e. both above 4-fold or 2-4-fold.

^d^ The final number of differential expressed genes were subtracted by number of overlapping genes indicated in parenthesis.

^e^ Number in parenthesis indicates the number of overlapping genes between early and late stationary phase with different fold change level, i.e. one above 4-fold and the other 2-4-fold.
