# Supplementary material for: Phenotypic and Genome-Wide Analysis of an Antibiotic-Resistant Small Colony Variant (SCV) of Pseudomonas aeruginosa
Source: PLoS One. 2011 Dec 15;6(12):e29276. doi: 10.1371/journal.pone.0029276 (PMC3240657; doi:10.1371/journal.pone.0029276)
Supplement: Table S2 — Functional classification of differentially expressed genes between P. aeruginosa PAO-SCV and its clonal wild-type PAO1 during stationary phase. (DOC) [file pone.0029276.s005.doc]

| **Table S2. Functional classification of differentially expressed genes between *P. aeruginosa* PAO-SCV and its clonal wild-type PAO1 during stationary phase** | | | | | | |
| --- | --- | --- | --- | --- | --- | --- |
|  |  |  |  |  |  |  |
| **Functional classification ^a^** | **Number of classified genes** | | | | | |
|  | **>4 fold** ^c^ | | **2-4 fold** | | **>2 fold** | |
|  | **SCV** | **WT** | **SCV** | **WT** | **SCV** | **WT** |
| Adaptation, protection | 2 | 0 | 5 | 2 | 7 | 2 |
| Amino acid biosynthesis and metabolism | 11 | 1 | 22 | 4 | 31 (-2 ^d^) | 5 |
| Antibiotic resistance and susceptibility | 2 | 0 | 4 | 2 | 5 (-1) | 2 |
| Biosynthesis of cofactors, prosthetic groups and carriers | 0 | 7 | 11 | 2 | 11 | 9 |
| Carbon compound catabolism | 8 | 3 | 9 | 2 | 17 | 5 |
| Cell division | 0 | 0 | 3 | 0 | 3 | 0 |
| Cell wall / LPS ^b^ / capsule | 6 | 0 | 7 | 6 | 11 (-2) | 6 |
| Central intermediary metabolism | 3 | 2 | 10 | 3 | 13 | 4 (-1) |
| Chaperones and heat shock proteins | 0 | 0 | 9 | 1 | 9 | 1 |
| Chemotaxis | 0 | 0 | 2 | 0 | 2 | 0 |
| DNA replication, recombination, modification and repair | 1 | 0 | 4 | 0 | 5 | 0 |
| Energy metabolism | 7 | 1 | 21 | 1 | 28 | 2 |
| Fatty acid and phospholipid metabolism | 0 | 0 | 6 | 2 | 6 | 2 |
| Hypothetical, unclassified, unknown | 21 | 14 | 123 | 55 | 137 (-7) | 62 (-7) |
| Membrane proteins | 8 | 0 | 20 | 10 | 27 (-1) | 10 |
| Motility and Attachment | 0 | 0 | 0 | 1 | 0 | 1 |
| Nucleotide biosynthesis and metabolism | 0 | 0 | 4 | 2 | 4 | 2 |
| Protein secretion/export apparatus | 1 | 0 | 7 | 1 | 8 | 1 |
| Putative enzymes | 3 | 2 | 21 | 10 | 25 (+1) | 11 (-1) |
| Related to phage, transposon, or plasmid | 0 | 4 | 0 | 24 | 0 | 27 (-1) |
| Secreted Factors (toxins, enzymes, alginate) | 1 | 4 | 6 | 5 | 7 | 9 |
| Transcription, RNA processing and degradation | 0 | 0 | 3 | 1 | 3 | 1 |
| Transcriptional regulators | 5 | 1 | 28 | 5 | 32 (-1) | 6 |
| Translation, post-translational modification, degradation | 4 | 1 | 34 | 1 | 38 | 2 |
| Transport of small molecules | 13 | 1 | 25 | 5 | 37 (-1) | 6 |
| Two-component regulatory systems | 0 | 0 | 0 | 0 | 0 | 0 |
| Non-coding RNA gene | 0 | 0 | 0 | 0 | 0 | 0 |
| **Total** | **96** | **41** | **384** | **145** | **466** | **176** |

^a^ Functional classification is from Pseudomonas Genome Database (http://www.pseudomonas.
com). Some minor modifications were arbitrarily made when we analyzed the gene expression data to minimize the overestimated numbers of classified genes. To be specific, genes belonging to group of two-component regulatory systems were removed into group of transcriptional regulators; Genes with hypothetical or unknown classification but with predicted membrane locations were changed to genes encoding membrane proteins.

^b^ LPS, lipopolysaccharide.

^c^ Genes with different fold-changes in expression were classified in PAO-SCV and wild type.

^d^ Number in parenthesis means the number of overlapping genes between two levels of fold change or adaption from recent annotations. Minus means subtraction; plus means addition from recent gene annotation.
