# Supplementary material for: Phenotypic and Genome-Wide Analysis of an Antibiotic-Resistant Small Colony Variant (SCV) of Pseudomonas aeruginosa
Source: PLoS One. 2011 Dec 15;6(12):e29276. doi: 10.1371/journal.pone.0029276 (PMC3240657; doi:10.1371/journal.pone.0029276)
Supplement: Table S3 — Up-regulated genes in P. aeruginosa PAO-SCV compared to its clonal wild-type PAO1 during stationary phase. (DOC) [file pone.0029276.s006.doc]

| **Table S3. Up-regulated genes in *P. aeruginosa* PAO-SCV compared to its clonal wild-type PAO1 during stationary phase** | | | | |
| --- | --- | --- | --- | --- |
|  |  |  |  |  |
| **PA number** | **Gene name** | **Fold change** | | **Pruduct name** |
|  |  | **Early SP** | **Late SP** |  |
| Adaptation, protection | | | |  |
| PA0867 | *mliC* |  | 8.1 | Membrane-bound lysozyme inhibitor of c-type lysozyme MliC |
| PA1008 | *bcp* |  | 2.9 | Bacterioferritin comigratory protein |
| PA2830 | *htpX* | 2.4 | 2.1 | Heat shock protein HtpX |
| PA3476 | *rhlI* |  | 2.1 | Autoinducer synthesis protein RhlI |
| PA4378 | *inaA* |  | 2.3 | InaA protein |
| PA4566 | *obg* |  | 2.0 | GTP-binding protein Obg |
| PA4614 | *mscL* |  | 6.2 | Conductance mechanosensitive channel |
|  |  |  |  |  |
| Amino acid biosynthesis and metabolism | | | |  |
| PA0036 | *trpB* | 3.0 |  | Tryptophan synthase beta chain |
| PA0609 | *trpE* | 2.1 |  | Anthranilate synthetase component I |
| PA0650 | *trpD* |  | 2.5 | Anthranilate phosphoribosyltransferase |
| PA0651 | *trpC* | 2.0 |  | Indole-3-glycerol-phosphate synthase |
| PA0865 | *hpd* | 4.7 |  | 4-Hydroxyphenylpyruvate dioxygenase |
| PA0870 | *phhC* | 2.4 |  | Aromatic amino acid aminotransferase |
| PA0895 | *aruC* |  | 2.4 | N-succinylglutamate 5-semialdehyde dehydrogenase |
| PA1326 | *ilvA2* | 5.4 |  | Threonine dehydratase, biosynthetic |
| PA1684 |  |  | 2.1 | 1,2-dihydroxy-3-keto-5-methylthiopentene dioxygenase MtnD |
| PA1999 | *dhcA* | 11.4 |  | Probable CoA transferase, subunit A |
| PA2081 | *kynB* |  | 2.2 | Kynurenine formamidase, KynB |
| PA2104 |  | 2.2 |  | Probable cysteine synthase |
| PA2247 | *bkdA1* |  | 4.1 | 2-oxoisovalerate dehydrogenase (alpha subunit) |
| PA2249 | *bkdB* |  | 5.0 | Branched-chain alpha-keto acid dehydrogenase (lipoamide component) |
| PA2444 | *glyA2* | 3.5 |  | Serine hydroxymethyltransferase |
| PA2445 | *gcvP2* | 3.8 |  | Glycine cleavage system protein P2 |
| PA2446 | *gcvH2* | 5.6 |  | Glycine cleavage system protein H2 |
| PA4565 | *proB* |  | 2.1 | Glutamate 5-kinase |
| PA4846 | *aroQ1* |  | 2.4 | 3-dehydroquinate dehydratase |
| PA4977 | *aruI* |  | 2.9 | 2-ketoarginine decarboxylase, AruI |
| PA5013 | *ilvE* |  | 2.4 | Branched-chain amino acid transferase |
| PA5143 | *hisB* | 2.1 |  | Imidazoleglycerol-phosphate dehydratase |
| PA5379 | *sdaB* |  | 2.2 | L-serine dehydratase |
| PA5398 | *dgcA* |  | 7.0 | DgcA, Dimethylglycine catabolism |
| PA5399 | *dgcB* |  | 3.4 | DgcB, Dimethylglycine catabolism |
| PA5410 | *gbcA* | 2.0 | 8.9 | GbcA |
| PA5411 | *gbcB* |  | 7.2 | GbcB |
| PA5415 | *glyA1* | 3.2 | 4.3 | Serine hydroxymethyltransferase |
| PA5416 | *soxB* |  | 7.2 | Sarcosine oxidase beta subunit |
| PA5417 | *soxD* |  | 3.8 | Sarcosine oxidase delta subunit |
| PA5419 | *soxG* |  | 3.1 | Sarcosine oxidase gamma subunit |
|  |  |  |  |  |
| Antibiotic resistance and susceptibility | | | |  |
| PA0425 | *mexA* | 3.3 | 2.2 | RND multidrug efflux protein MexA precursor |
| PA0427 | *oprM* | 2.4 |  | Outer membrane protein OprM precursor |
| PA0706 | *cat* | 2.9 | 4.5 | Chloramphenicol acetyltransferase |
| PA2018 | *mexY* | 3.9 |  | RND multidrug efflux transporter |
| PA2019 | *mexX* | 8.7 |  | RND multidrug efflux membrane fusion protein precursor |
|  |  |  |  |  |
| Biosynthesis of cofactors, prosthetic groups and carriers | | | | |
| PA0500 | *bioB* | 3.2 | 3.8 | Biotin synthase |
| PA0501 | *bioF* | 2.6 |  | 8-amino-7-oxononanoate synthase |
| PA0672 | *hemO* |  | 2.4 | Heme oxygenase |
| PA1674 | *folE2* | 2.3 |  | GTP cyclohydrolase I precursor |
| PA1772 | *menG* |  | 2.1 | Probable methyltransferase |
| PA1796 | *folD* | 2.1 |  | 5,10-methylene-tetrahydrofolate dehydrogenase/cyclohydrolase |
| PA1985 | *pqqA* | 3.0 |  | Pyrroloquinoline quinone biosynthesis protein A |
| PA1986 | *pqqB* |  | 2.4 | Pyrroloquinoline quinone biosynthesis protein B |
| PA1987 | *pqqC* |  | 2.4 | Pyrroloquinoline quinone biosynthesis protein C |
| PA3029 | *moaB2* |  | 2.8 | Molybdopterin biosynthetic protein B2 |
| PA3439 | *folX* |  | 2.8 | d-erythro-7,8-dihydroneopterin triphosphate epimerase |
|  |  |  |  |  |
| Carbon compound catabolism | | |  |  |
| PA0795 | *prpC* |  | 4.9 | Citrate synthase 2 |
| PA0796 | *prpB* |  | 3.6 | Carboxyphosphonoenolpyruvate phosphonomutase |
| PA1770 | *ppsA* |  | 4 | Phosphoenolpyruvate synthase |
| PA1950 | *rbsK* |  | 2.2 | Ribokinase |
| PA1982 | *exaA* |  | 14.2 | Quinoprotein alcohol dehydrogenase |
| PA2003 | *bdhA* | 5.2 |  | 3-hydroxybutyrate dehydrogenase |
| PA2007 | *maiA* | 5.5 |  | Maleylacetoacetate isomerase |
| PA2011 | *liuE* | 2.3 |  | 3-hydroxy-3-methylglutaryl-CoA lyase |
| PA2015 | *liuA* |  | 3.5 | Putative isovaleryl-CoA dehydrogenase |
| PA2796 | *tal* |  | 2.4 | Transaldolase |
| PA3183 | *zwf* | 2.3 |  | Glucose-6-phosphate 1-dehydrogenase |
| PA3195 | *gapA* |  | 4.5 | Glyceraldehyde 3-phosphate dehydrogenase |
| PA3366 | *amiE* |  | 4.8 | Aliphatic amidase |
| PA3570 | *mmsA* |  | 4.3 | Methylmalonate-semialdehyde dehydrogenase |
| PA3710 |  | 3.1 |  | Probable GMC-type oxidoreductase |
| PA4733 | *acsB* | 2.2 | 4 | Acetyl-coenzyme A synthetase |
| PA5418 | *soxA* |  | 2.7 | Sarcosine oxidase alpha subunit |
|  |  |  |  |  |
| Cell division | |  |  |  |
| PA1528 | *zipA* |  | 2.3 | cell division protein ZipA |
| PA3244 | *minD* |  | 2.4 | cell division inhibitor MinD |
| PA3245 | *minE* |  | 3.0 | cell division topological specificity factor MinE |
|  |  |  |  |  |
| Cell wall / LPS / capsule | |  |  |  |
| PA0705 | *migA* | 6.4 | 3.6 | Alpha-1,6-rhamnosyltransferase MigA |
| PA3552 | *arnB* | 13.8 | 24.4 | ArnB |
| PA3553 | *arnC* | 7.6 | 10.8 | ArnC |
| PA3554 | *arnA* |  | 9.8 | ArnA |
| PA3555 | *arnD* | 3.2 |  | ArnD |
| PA3556 | *arnT* | 4.2 | 3.9 | Inner membrane L-Ara4N transferase ArnT |
| PA3557 | *arnE* | 2.5 | 2.7 | ArnE |
| PA3558 | *arnF* | 4.1 | 5.4 | ArnF |
| PA4997 | *msbA* | 2.1 |  | Transport protein MsbA |
| PA5276 | *lppL* |  | 3.0 | Lipopeptide LppL precursor |
| PA5450 | *wzt* |  | 2.0 | ABC subunit of A-band LPS efflux transporter |
|  |  |  |  |  |
| Central intermediary metabolism | | |  |  |
| PA0102 |  | 2.4 | 3.4 | Probable carbonic anhydrase |
| PA2001 | *atoB* | 8.3 |  | Acetyl-CoA acetyltransferase |
| PA2023 | *galU* |  | 2.3 | UTP--glucose-1-phosphate uridylyltransferase |
| PA3471 | *sfcA* |  | 3.3 | Probable malic enzyme |
| PA3524 | *gloA1* |  | 3.2 | Lactoylglutathione lyase |
| PA3629 | *adhC* |  | 3.9 | Alcohol dehydrogenase class III |
| PA4024 | *eutB* | 2.7 |  | Ethanolamine ammonia-lyase large subunit |
| PA4031 | *ppa* |  | 2.1 | Inorganic pyrophosphatase |
| PA4443 | *cysD* |  | 2.1 | ATP sulfurylase small subunit |
| PA4733 | *acsB* | 2.2 | 4.0 | Acetyl-coenzyme A synthetase |
| PA4880 |  |  | 3.5 | Probable bacterioferritin |
| PA5056 | *phaC1* |  | 2.3 | Poly(3-hydroxyalkanoic acid) synthase 1 |
| PA5421 | *fdhA* |  | 4.4 | Glutathione-independent formaldehyde dehydrogenase |
|  |  |  |  |  |
| Chaperones and heat shock proteins | | | |  |
| PA0837 | *slyD* |  | 2.9 | Peptidyl-prolyl cis-trans isomerase SlyD |
| PA1800 | *tig* |  | 2.2 | Trigger factor |
| PA1805 | *ppiD* |  | 2.5 | Peptidyl-prolyl cis-trans isomerase D |
| PA3262 |  | 2.0 | 2.3 | Probable peptidyl-prolyl cis-trans isomerase, FkbP-type |
| PA3365 | *amiB* | 3.7 | 2.6 | Probable chaperone |
| PA3737 | *dsbC* |  | 2.4 | Thiol:disulfide interchange protein DsbC |
| PA5129 | *grx* |  | 2.2 | Glutaredoxin |
| PA5240 | *trxA* |  | 2.2 | Thioredoxin |
| PA5489 | *dsbA* |  | 2.1 | Thiol:disulfide interchange protein DsbA |
|  |  |  |  |  |
| Chemotaxis | |  |  |  |
| PA4915 |  |  | 3.1 | Probable chemotaxis transducer |
| PA4953 | *motB* |  | 2.1 | Chemotaxis protein MotB |
|  |  |  |  |  |
| DNA replication, recombination, modification and repair | | | | |
| PA0669 |  |  | 2.4 | Probable DNA polymerase alpha chain |
| PA3940 |  |  | 2.6 | Probable DNA binding protein |
| PA4232 | *ssb* |  | 2.9 | Single-stranded DNA-binding protein |
| PA4281 | *sbcD* | 2.3 |  | Exonuclease SbcD |
| PA5348 |  |  | 8.4 | Probable DNA-binding protein |
|  |  |  |  |  |
| Energy metabolism | |  |  |  |
| PA0105 | *coxB* |  | 3.4 | Cytochrome c oxidase, subunit II |
| PA0106 | *coxA* |  | 6.2 | Cytochrome c oxidase, subunit I |
| PA0107 |  |  | 3.4 | Conserved hypothetical protein |
| PA0108 | *coIII* |  | 5.7 | Cytochrome c oxidase, subunit III |
| PA0113 |  |  | 2.6 | Probable cytochrome c oxidase assembly factor |
| PA0589 |  |  | 2.1 | Conserved hypothetical protein |
| PA1175 | *napD* |  | 4.2 | NapD protein of periplasmic nitrate reductase |
| PA1176 | *napF* |  | 2.5 | Ferredoxin protein NapF |
| PA1177 | *napE* |  | 3.5 | Periplasmic nitrate reductase protein NapE |
| PA1554 | *glpE* |  | 2.7 | Probable cytochrome oxidase subunit (cbb3-type) |
| PA1580 | *gltA* |  | 4.4 | Citrate synthase |
| PA1581 | *sdhC* |  | 3.1 | Succinate dehydrogenase (C subunit) |
| PA1582 | *sdhD* |  | 3.5 | Succinate dehydrogenase (D subunit) |
| PA1584 | *sdhB* |  | 2.3 | Succinate dehydrogenase (B subunit) |
| PA1588 | *sucC* |  | 4.6 | Succinyl-CoA synthetase beta chain |
| PA1983 | *exaB* |  | 25.7 | Cytochrome c550 |
| PA2623 | *icd* |  | 4.0 | Isocitrate dehydrogenase |
| PA2640 | *nuoE* |  | 3.7 | NADH dehydrogenase I chain E |
| PA2951 | *etfA* |  | 2.6 | Electron transfer flavoprotein alpha-subunit |
| PA3621 | *fdxA* |  | 2.7 | Ferredoxin I |
| PA3930 | *cioA* |  | 2.7 | Cyanide insensitive terminal oxidase |
| PA4430 |  |  | 3.1 | Probable cytochrome b |
| PA4812 | *fdnG* | 2.2 |  | Formate dehydrogenase-O, major subunit |
| PA5300 | *cycB* |  | 2.3 | Cytochrome c5 |
| PA5400 |  |  | 2.1 | Probable electron transfer flavoprotein alpha subunit |
| PA5490 | *cc4* |  | 4.3 | Cytochrome c4 precursor |
| PA5559 | *atpE* |  | 2.3 | ATP synthase C chain |
| PA5560 | *atpB* |  | 2.0 | ATP synthase A chain |
|  |  |  |  |  |
| Fatty acid and phospholipid metabolism | | | |  |
| PA0447 | *gcdH* | 3.4 |  | Glutaryl-CoA dehydrogenase |
| PA2557 |  |  | 2.1 | Probable AMP-binding enzyme |
| PA2966 | *acpP* |  | 2.6 | Acyl carrier protein |
| PA3300 | *fadD2* |  | 2.2 | Long-chain-fatty-acid--CoA ligase |
| PA3603 | *dgkA* | 2.2 |  | Diacylglycerol kinase |
| PA3924 |  |  | 2.2 | Probable medium-chain acyl-CoA ligase |
|  |  |  |  |  |
| Membrane proteins | |  |  |  |
| PA0112 |  |  | 2.0 | Hypothetical protein |
| PA0702 |  |  | 2.2 | Hypothetical protein |
| PA0833 |  | 4.3 | 3.3 | Hypothetical protein |
| PA1053 | *slyB* |  | 3.0 | Conserved hypothetical protein |
| PA1178 | *oprH* | 4.3 | 5.1 | PhoP/Q and low Mg2+ inducible outer membrane protein H1 precursor |
| PA1190 | *yohC* |  | 3.8 | Conserved hypothetical protein |
| PA1288 | *ompP1*/*fadL* |  | 3.1 | Probable outer membrane protein precursor |
| PA1676 |  | 2.2 |  | Hypothetical protein |
| PA2002 | *atoE* | 5.3 |  | Conserved hypothetical protein |
| PA2004 |  | 6.2 |  | Conserved hypothetical protein |
| PA2746 |  |  | 4.9 | Hypothetical protein |
| PA2946 |  | 2.3 |  | Hypothetical protein |
| PA3079 |  |  | 2.0 | Hypothetical protein |
| PA3310 |  | 2.6 | 2.4 | Conserved hypothetical protein |
| PA3369 |  |  | 2.6 | Hypothetical protein |
| PA3575 |  |  | 2.4 | Hypothetical protein |
| PA3819 | *ycfJ* |  | 5.5 | Conserved hypothetical protein |
| PA4011 |  | 4.1 | 5.5 | Hypothetical protein |
| PA4370 | *icmP* | 2.2 |  | Insulin-cleaving metalloproteinase outer membrane protein precursor |
| PA4455 | *yrbE* | 2.1 |  | Probable permease of ABC transporter |
| PA4501 | *opdD* |  | 2.2 | Glycine-glutamate dipeptide porin OpdP |
| PA4765 | *omlA* |  | 2.7 | Outer membrane lipoprotein OmlA precursor |
| PA4912 |  |  | 2.1 | Probable permease of ABC branched chain amino acid transporter |
| PA5183 |  |  | 2.3 | Hypothetical protein |
| PA5250 |  |  | 3.4 | Conserved hypothetical protein |
| PA5424 | *yeaQ* | 2.5 | 2.7 | Conserved hypothetical protein |
| PA5528 |  |  | 4.2 | Hypothetical protein |
|  |  |  |  |  |
| Nucleotide biosynthesis and metabolism | | | |  |
| PA0441 | *dht* |  | 2.0 | Dihydropyrimidinase |
| PA0444 | *hyuC* |  | 2.3 | N-carbamoyl-beta-alanine amidohydrolase |
| PA2616 | *trxB1* |  | 2.4 | Thioredoxin reductase 1 |
| PA5298 | *xpt* |  | 2.3 | Xanthine phosphoribosyltransferase |
|  |  |  |  |  |
| Protein secretion/export apparatus | | |  |  |
| PA0677 | *hxcW* |  | 2.6 | HxcW putative pseudopilin |
| PA0680 | *hxcV* |  | 2.4 | HxcV putative pseudopilin |
| PA0683 | *hxcY* |  | 2.7 | Probable type II secretion system protein |
| PA0685 | *hxcQ* |  | 4.7 | Probable type II secretion system protein |
| PA0686 | *hxcR* |  | 2.2 | Probable type II secretion system protein |
| PA4403 | *secA* |  | 3.9 | Secretion protein SecA |
| PA4747 | *secG* |  | 2.0 | Secretion protein SecG |
| PA5128 | *secB* |  | 2.4 | Secretion protein SecB |
|  |  |  |  |  |
| Putative enzymes | |  |  |  |
| PA0296 | *spuI* | 3.9 | 2.9 | Probable glutamine synthetase |
| PA0298 | *spuB* | 3.4 | 3.3 | Probable glutamine synthetase |
| PA0372 |  | 2.2 |  | Probable zinc protease |
| PA0473 | *psfA* |  | 2.6 | Probable glutathione S-transferase |
| PA0704 |  |  | 4.9 | Probable amidase |
| PA0745 |  |  | 3.4 | Probable enoyl-CoA hydratase/isomerase |
| PA0779 |  | 2.1 |  | Probable ATP-dependent protease |
| PA1296 |  | 2.1 |  | Probable 2-hydroxyacid dehydrogenase |
| PA1344 | *yvaG* | 2.4 |  | Probable short-chain dehydrogenase |
| PA1617 |  |  | 2.1 | Probable AMP-binding enzyme |
| PA1984 | *exaC1* |  | 7.8 | Probable aldehyde dehydrogenase |
| PA1991 |  | 2.9 | 2.7 | Probable iron-containing alcohol dehydrogenase |
| PA2379 |  |  | 3.0 | Probable oxidoreductase |
| PA2634 | *aceA* |  | 2.2 | Isocitrate lyase AceA |
| PA3001 |  |  | 2.3 | Probable glyceraldehyde-3-phosphate dehydrogenase |
| PA3295 |  |  | 3.0 | Probable HIT family protein |
| PA3559 |  | 7.4 | 8.5 | Probable nucleotide sugar dehydrogenase |
| PA3568 | *ymmS* |  | 3.1 | Probable acetyl-coa synthetase |
| PA4171 |  |  | 2.4 | Probable protease |
| PA4204 | *ppgL* | 2.5 |  | Periplasmic gluconolactonase PpgL |
| PA4425 | *yraO* |  | 3.7 | Sedoheptulose 7-phosphate isomerase GmhA |
| PA4431 |  |  | 2.8 | Probable iron-sulfur protein |
| PA4434 |  | 2.3 |  | Probable oxidoreductase |
| PA5313 | *paaT* |  | 2.2 | Probable pyridoxal-dependent aminotransferase |
| PA5521 |  | 2.2 |  | Probable short-chain dehydrogenase |
|  |  |  |  |  |
| Secreted Factors (toxins, enzymes, alginate) | | | | |
| PA2862 | *lipA* |  | 4.9 | Lactonizing lipase precursor |
| PA4221 | *fptA* | 2.7 |  | Fe(III)-pyochelin outer membrane receptor precursor |
| PA4226 | *pchE* | 2.8 |  | Dihydroaeruginoic acid synthetase PchE |
| PA4228 | *pchD* | 3.8 |  | Pyochelin biosynthesis protein PchD |
| PA4229 | *pchC* | 2.5 |  | Pyochelin biosynthetic protein PchC |
| PA4230 | *pchB* | 3.7 |  | Salicylate biosynthesis protein PchB |
| PA5112 | *estA* |  | 2.4 | Esterase EstA |
|  |  |  |  |  |
| Transcription, RNA processing and degradation | | | | |
| PA3161 | *himD* |  | 2.3 | Integration host factor beta subunit |
| PA4944 | *hfq* |  | 2.8 | Sm-like RNA-binding protein Hfq |
| PA5337 | *rpoZ* | 2.1 |  | RNA polymerase omega subunit |
|  |  |  |  |  |
| Transcriptional regulators | |  |  |  |
| PA0167 |  | 2.2 |  | Probable transcriptional regulator |
| PA0424 | *mexR* | 9.2 |  | Multidrug resistance operon repressor MexR |
| PA0676 |  |  | 3.3 | Probable transmembrane sensor |
| PA0701 |  |  | 2.0 | Probable transcriptional regulator |
| PA0707 | *toxR* |  | 2.5 | Transcriptional regulator ToxR |
| PA0762 | *algU* | 2.3 |  | Sigma factor AlgU |
| PA0763 | *mucA* | 2.0 |  | Anti-sigma factor MucA |
| PA0780 | *pruR* | 2.2 |  | Proline utilization regulator |
| PA0797 |  | 2.5 |  | Probable transcriptional regulator |
| PA0876 |  | 2.0 |  | Probable transcriptional regulator |
| PA0929 | *pirR* | 2.2 |  | Two-component response regulator |
| PA1159 |  |  | 4.8 | Probable cold-shock protein |
| PA1179 | *phoP* | 6.8 | 14.3 | Two-component response regulator PhoP |
| PA1180 | *phoQ* | 3.7 | 4.5 | Two-component sensor PhoQ |
| PA1504 |  | 2.5 |  | Probable transcriptional regulator |
| PA1754 | *cysB* |  | 3.4 | Transcriptional regulator CysB |
| PA1949 | *rbsR* |  | 2.0 | Ribose operon repressor RbsR |
| PA1978 | *agmR* |  | 6.9 | Probable transcriptional regulator |
| PA2020 | *amrR/mexZ* | 3.4 | 2.5 | Probable transcriptional regulator |
| PA2388 | *fpvR* | 2.4 |  | Probable transmembrane sensor |
| PA2622 | *cspD* |  | 3.8 | Cold-shock protein CspD |
| PA2896 |  | 2.2 |  | Probable sigma-70 factor, ECF subfamily |
| PA3385 | *amrZ* | 2.1 |  | Alginate and motility regulator Z |
| PA3622 | *rpoS* |  | 4.0 | Sigma factor RpoS |
| PA4723 | *dksA* |  | 2.3 | Suppressor protein DksA |
| PA4764 | *fur* |  | 2.7 | Ferric uptake regulation protein |
| PA5253 | *algP* |  | 3.2 | Alginate regulatory protein AlgP |
| PA5255 | *algQ* |  | 2.2 | Alginate regulatory protein AlgQ |
| PA5301 | *ycjC* | 2.5 | 2.7 | Probable transcriptional regulator |
| PA5360 | *phoB* |  | 2.2 | Two-component response regulator PhoB |
| PA5380 | *gbdR* |  | 3.0 | AraC family transcription factor GbdR |
| PA5484 | *kinB* | 2.1 |  | Probable two-component sensor |
|  |  |  |  |  |
| Translation, post-translational modification, degradation | | | | |
| PA0019 | *def* |  | 2.1 | Polypeptide deformylase |
| PA1122 | *fms*/*pdf*/*def* | 2.1 | 2.5 | Probable peptide deformylase |
| PA2071 | *fusA2* | 2.1 |  | Elongation factor G |
| PA2612 | *serS* | 2.4 |  | Seryl-tRNA synthetase |
| PA2619 | *infA* | 2.5 | 3.0 | Initiation factor |
| PA2620 | *clpA* | 2.4 |  | ATP-binding protease component ClpA |
| PA2739 | *pheT* | 2.3 |  | Phenylalanyl-tRNA synthetase, beta subunit |
| PA2740 | *pheS* | 2.9 |  | Phenylalanyl-tRNA synthetase, alpha-subunit |
| PA2742 | *rpmI* |  | 4.4 | 50S ribosomal protein L35 |
| PA2755 | *eco* |  | 2.3 | Ecotin precursor |
| PA2851 | *efp* |  | 3.5 | Translation elongation factor P |
| PA3049 | *rmf* |  | 2.7 | Ribosome modulation factor |
| PA3162 | *rpsA* |  | 2.5 | 30S ribosomal protein S1 |
| PA3834 | *valS* |  | 2.1 | Valyl-tRNA synthetase |
| PA3987 | *leuS* |  | 2.7 | Leucyl-tRNA synthetase |
| PA4138 | *tyrS* | 3.1 |  | Tyrosyl-tRNA synthetase |
| PA4242 | *rpmJ* |  | 8.6 | 50S ribosomal protein L36 |
| PA4245 | *rpmD* |  | 2.7 | 50S ribosomal protein L30 |
| PA4246 | *rpsE* |  | 3.3 | 30S ribosomal protein S5 |
| PA4247 | *rplR* |  | 3.4 | 50S ribosomal protein L18 |
| PA4248 | *rplF* |  | 2.1 | 50S ribosomal protein L6 |
| PA4249 | *rpsH* |  | 2.0 | 30S ribosomal protein S8 |
| PA4252 | *rplX* |  | 3.8 | 50S ribosomal protein L24 |
| PA4253 | *rplN* |  | 2.5 | 50S ribosomal protein L14 |
| PA4254 | *rpsQ* |  | 3.2 | 30S ribosomal protein S17 |
| PA4262 | *rplD* |  | 2.9 | 50S ribosomal protein L4 |
| PA4264 | *rpsJ* |  | 3.2 | 30S ribosomal protein S10 |
| PA4265 | *tufA* |  | 2.7 | Elongation factor Tu |
| PA4268 | *rpsL* |  | 2.8 | 30S ribosomal protein S12 |
| PA4274 | *rplK* |  | 2.6 | 50S ribosomal protein L11 |
| PA4433 | *rplM* |  | 6.7 | 50S ribosomal protein L13 |
| PA4563 | *rpsT* |  | 3.0 | 30S ribosomal protein S20 |
| PA4567 | *rpmA* |  | 2.0 | 50S ribosomal protein L27 |
| PA4568 | *rplU* |  | 4.2 | 50S ribosomal protein L21 |
| PA4671 | *rplY* |  | 2.0 | Probable ribosomal protein L25 |
| PA4935 | *rpsF* |  | 2.7 | 30S ribosomal protein S6 |
| PA5049 | *rpmE* |  | 3.0 | 50S ribosomal protein L31 |
| PA5316 | *rpmB* |  | 3.4 | 50S ribosomal protein L28 |
|  |  |  |  |  |
| Transport of small molecules | | |  |  |
| PA0300 | *spuD* |  | 3.5 | Polyamine transport protein PotF2 |
| PA0302 | *spuF* |  | 2.1 | Polyamine transport protein PotG |
| PA0688 |  |  | 2.4 | Probable binding protein component of ABC transporter |
| PA0693 | *exbB2* |  | 3.0 | Transport protein ExbB2 |
| PA0694 | *exbD2* |  | 2.1 | Transport protein ExbD |
| PA0703 |  |  | 2.2 | Probable major facilitator superfamily (MFS) transporter |
| PA0866 | *aroP2* | 4.6 |  | Aromatic amino acid transport protein AroP2 |
| PA0958 | *oprD* |  | 5.0 | Basic amino acid, basic peptide and imipenem outer membrane porin OprD precursor |
| PA0972 | *tolB* | 2.2 |  | TolB protein |
| PA1070 | *braG* |  | 2.5 | Branched-chain amino acid transport protein BraG |
| PA1074 | *braC* |  | 3.5 | Branched-chain amino acid transport protein BraC |
| PA1342 |  |  | 3.0 | Probable binding protein component of ABC transporter |
| PA1493 | *cysP* |  | 2.5 | Sulfate-binding protein of ABC transporter |
| PA1946 | *rbsB* |  | 8.6 | Binding protein component precursor of ABC ribose transporter |
| PA1947 | *rbsA* |  | 4.2 | Ribose transport protein RbsA |
| PA1948 | *rbsC* |  | 2.2 | Membrane protein component of ABC ribose transporter |
| PA2041 |  | 2.8 |  | Probable amino acid permease |
| PA2760 | *oprQ* |  | 6.1 | Probable outer membrane protein precursor |
| PA3038 | *opdQ* |  | 7.1 | Probable porin |
| PA3186 | *oprB* |  | 6.4 | Glucose/carbohydrate outer membrane porin OprB precursor |
| PA3187 | *gltK* |  | 3.0 | Probable ATP-binding component of ABC transporter |
| PA3188 | *gltG* |  | 3.2 | Probable permease of ABC sugar transporter |
| PA3190 | *gltB* |  | 4.4 | Probable binding protein component of ABC sugar transporter |
| PA3234 | *yjcG* | 5.6 | 2.5 | Probable sodium:solute symporter |
| PA3236 |  |  | 10.6 | Probable glycine betaine-binding protein precursor |
| PA3889 |  | 2.1 |  | Probable binding protein component of ABC transporter |
| PA4023 | *eutP* | 2.3 |  | Probable transport protein |
| PA4456 | *yrbF* | 2.3 |  | Probable ATP-binding component of ABC transporter |
| PA4461 | *yhbG* |  | 2.3 | Probable ATP-binding component of ABC transporter |
| PA4496 |  |  | 3.3 | Probable binding protein component of ABC transporter |
| PA4500 |  |  | 10.6 | Probable binding protein component of ABC transporter |
| PA4502 |  |  | 3.1 | Probable binding protein component of ABC transporter |
| PA4913 |  |  | 2.2 | Probable binding protein component of ABC transporter |
| PA5152 |  |  | 2.9 | Probable ATP-binding component of ABC transporter |
| PA5153 |  |  | 8.0 | Probable periplasmic binding protein |
| PA5167 |  |  | 4.2 | Probable c4-dicarboxylate-binding protein |
| PA5376 |  |  | 2.6 | Probable ATP-binding component of ABC transporter |
|  |  |  |  |  |
| Hypothetical, unclassified, unknown | | | |  |
| PA0039 |  |  | 3.8 | Hypothetical protein |
| PA0068 |  | 2.3 |  | Hypothetical protein |
| PA0114 |  |  | 3.4 | Conserved hypothetical protein |
| PA0315 |  |  | 2.8 | Hypothetical protein |
| PA0329 |  |  | 2.3 | Conserved hypothetical protein |
| PA0384 |  |  | 2.3 | Hypothetical protein |
| PA0446 |  | 3.7 |  | Conserved hypothetical protein |
| PA0449 |  | 2.1 |  | Hypothetical protein |
| PA0461 | *yihG* |  | 2.4 | Conserved hypothetical protein |
| PA0537 |  |  | 2.0 | Conserved hypothetical protein |
| PA0655 |  |  | 2.1 | Hypothetical protein |
| PA0667 | *yebA* | 2.2 | 2.5 | Conserved hypothetical protein |
| PA0670 |  |  | 2.8 | Hypothetical protein |
| PA0671 |  |  | 2.2 | Hypothetical protein |
| PA0673 | *pigB* |  | 3.4 | Hypothetical protein |
| PA0691 |  |  | 2.2 | Hypothetical protein |
| PA0692 |  |  | 2.2 | Hypothetical protein |
| PA0696 |  |  | 2.3 | Hypothetical protein |
| PA0697 |  |  | 2.2 | Hypothetical protein |
| PA0698 |  |  | 3.5 | Hypothetical protein |
| PA0700 |  |  | 3.5 | Hypothetical protein |
| PA0805 |  | 2.3 | 4.1 | Hypothetical protein |
| PA0856 |  | 3.0 | 3.5 | Hypothetical protein |
| PA0900 |  |  | 4.5 | Hypothetical protein |
| PA0919 |  | 2.1 |  | Hypothetical protein |
| PA0943 |  | 2.3 | 2.3 | Hypothetical protein |
| PA0960 |  |  | 3.3 | Hypothetical protein |
| PA0974 |  | 2.3 |  | Conserved hypothetical protein |
| PA1009 |  | 2.6 |  | Hypothetical protein |
| PA1106 |  | 2.2 |  | Hypothetical protein |
| PA1189 |  | 2.0 |  | Conserved hypothetical protein |
| PA1198 |  | 2.2 | 4.3 | Conserved hypothetical protein |
| PA1323 |  | 5.8 | 2.1 | Hypothetical protein |
| PA1324 |  |  | 2.7 | Hypothetical protein |
| PA1325 | *yybH* | 3.9 |  | Conserved hypothetical protein |
| PA1333 |  | 2.1 | 2.6 | Hypothetical protein |
| PA1353 |  |  | 2.0 | Hypothetical protein |
| PA1354 |  |  | 2.1 | Hypothetical protein |
| PA1404 |  |  | 2.6 | Hypothetical protein |
| PA1471 |  | 6.4 | 3.3 | Hypothetical protein |
| PA1494 |  | 2.2 |  | Conserved hypothetical protein |
| PA1533 |  |  | 2.2 | Conserved hypothetical protein |
| PA1571 |  |  | 6.8 | Hypothetical protein |
| PA1574 | *yaiE* |  | 6.7 | Conserved hypothetical protein |
| PA1579 |  |  | 4.2 | Hypothetical protein |
| PA1616 | *sixA* | 2.3 |  | Conserved hypothetical protein |
| PA1618 | *ybdB* | 2.5 |  | Conserved hypothetical protein |
| PA1640 |  | 2.6 |  | Conserved hypothetical protein |
| PA1688 |  | 3.2 |  | Hypothetical protein |
| PA1728 |  |  | 2.7 | Hypothetical protein |
| PA1745 |  |  | 2.5 | Hypothetical protein |
| PA1747 |  | 2.4 | 2.9 | Hypothetical protein |
| PA1761 |  | 2.6 | 3.0 | Hypothetical protein |
| PA1765 |  |  | 2.5 | Hypothetical protein |
| PA1830 |  |  | 2.9 | Hypothetical protein |
| PA1852 |  |  | 4.6 | Hypothetical protein |
| PA1942 |  |  | 4.1 | Hypothetical protein |
| PA1944 |  | 2.8 |  | Hypothetical protein |
| PA1963 |  | 2.1 | 2.2 | Hypothetical protein |
| PA1968 |  |  | 2.7 | Hypothetical protein |
| PA1969 |  | 2.1 |  | Hypothetical protein |
| PA1974 |  |  | 2.5 | Hypothetical protein |
| PA2044 |  | 2.5 |  | Hypothetical protein |
| PA2080 | *kynU* |  | 2.4 | Kynureninase KynU |
| PA2146 | *yciG* | 4.8 |  | Conserved hypothetical protein |
| PA2159 |  |  | 2.0 | Conserved hypothetical protein |
| PA2166 |  |  | 3.2 | Hypothetical protein |
| PA2171 |  |  | 3.1 | Hypothetical protein |
| PA2223 |  |  | 2.2 | Hypothetical protein |
| PA2441 |  | 2.1 |  | Hypothetical protein |
| PA2485 |  | 4.7 |  | Hypothetical protein |
| PA2504 |  |  | 2.4 | Hypothetical protein |
| PA2562 |  | 2.6 | 2.8 | Hypothetical protein |
| PA2569 |  | 2.2 |  | Hypothetical protein |
| PA2659 |  |  | 2.8 | Hypothetical protein |
| PA2667 |  | 2.3 | 6.1 | Conserved hypothetical protein |
| PA2707 |  |  | 2.3 | Hypothetical protein |
| PA2747 |  |  | 2.6 | Hypothetical protein |
| PA2762 |  |  | 7.3 | Hypothetical protein |
| PA2779 |  | 3.7 | 3.4 | Hypothetical protein |
| PA2971 | *yceD* |  | 2.1 | Conserved hypothetical protein |
| PA3031 |  | 3.9 | 3.3 | Hypothetical protein |
| PA3179 | *yciL* |  | 2.0 | Conserved hypothetical protein |
| PA3224 |  |  | 2.6 | Hypothetical protein |
| PA3238 |  | 3.0 |  | Hypothetical protein |
| PA3250 |  |  | 2.7 | Hypothetical protein |
| PA3440 |  |  | 2.8 | Conserved hypothetical protein |
| PA3470 |  |  | 2.0 | Hypothetical protein |
| PA3472 |  | 3.1 | 2.5 | Hypothetical protein |
| PA3496 |  | 2.6 | 3.8 | Hypothetical protein |
| PA3530 | *bfd* | 2.9 |  | Conserved hypothetical protein |
| PA3533 | *ydhD* |  | 3.5 | Conserved hypothetical protein |
| PA3674 |  |  | 2.6 | Hypothetical protein |
| PA3836 |  |  | 2.6 | Hypothetical protein |
| PA3848 |  |  | 2.2 | Hypothetical protein |
| PA3922 |  |  | 3.4 | Conserved hypothetical protein |
| PA3923 |  |  | 5.6 | Hypothetical protein |
| PA3962 |  | 2.3 |  | Hypothetical protein |
| PA3981 | *ybeZ* | 2.4 |  | Conserved hypothetical protein |
| PA3988 |  |  | 2.4 | Hypothetical protein |
| PA4010 |  | 2.5 | 3.3 | Hypothetical protein |
| PA4015 |  |  | 2.3 | Conserved hypothetical protein |
| PA4154 | *ygiM* | 2.2 |  | Conserved hypothetical protein |
| PA4336 |  |  | 2.7 | Conserved hypothetical protein |
| PA4377 |  | 2.3 | 3.5 | Hypothetical protein |
| PA4379 |  |  | 4.5 | Conserved hypothetical protein |
| PA4395 | *yajQ* |  | 3.6 | Conserved hypothetical protein |
| PA4423 | *yraM* |  | 2.4 | Conserved hypothetical protein |
| PA4426 | *yraP* |  | 3.9 | Conserved hypothetical protein |
| PA4451 | *yrbA* | 2.0 |  | Conserved hypothetical protein |
| PA4453 |  |  | 3.4 | Conserved hypothetical protein |
| PA4454 | *yrbD* | 2.3 | 2.4 | Conserved hypothetical protein |
| PA4578 |  |  | 3.5 | Hypothetical protein |
| PA4625 |  | 2.7 |  | Hypothetical protein |
| PA4648 |  |  | 2.0 | Hypothetical protein |
| PA4684 |  | 2.5 |  | Hypothetical protein |
| PA4685 |  | 4.0 | 2.5 | Hypothetical protein |
| PA4686 |  | 2.1 |  | Hypothetical protein |
| PA4703 |  |  | 3.3 | Hypothetical protein |
| PA4714 |  |  | 2.7 | Conserved hypothetical protein |
| PA4739 |  | 11.4 |  | Conserved hypothetical protein |
| PA4793 |  |  | 2.2 | Hypothetical protein |
| PA4877 |  |  | 3.6 | Hypothetical protein |
| PA4972 |  | 2.1 |  | Hypothetical protein |
| PA5028 |  |  | 2.3 | Conserved hypothetical protein |
| PA5101 |  |  | 2.5 | Hypothetical protein |
| PA5180 | *fdhD* |  | 2.4 | Conserved hypothetical protein |
| PA5191 |  |  | 3.1 | Hypothetical protein |
| PA5212 |  |  | 3.0 | Hypothetical protein |
| PA5233 |  |  | 3.3 | Hypothetical protein |
| PA5378 |  |  | 5.2 | Hypothetical protein |
| PA5396 |  | 3.2 | 14.2 | Hypothetical protein |
| PA5397 |  |  | 6.4 | Hypothetical protein |
| PA5461 |  |  | 6.9 | Hypothetical protein |
| PA5463 |  | 2.1 |  | Hypothetical protein |
| PA5526 |  | 2.5 | 3.1 | Hypothetical protein |
| PA5545 |  |  | 2.4 | Conserved hypothetical protein |

*^a^* PA number, gene name and product name are identified through Pseudomonas Genome Database (http://www.pseudomonas.com).

*^b^*  Selected genes with significant expression changes in a magnitude of at least 2-fold are listed (*P* value less than 0.05). SP, stationary phase.

*^c^* RND, resistance-nodulation-cell division; ABC, ATP-binding cassette; MFS, major facilitator superfamily.
