# Supplementary material for: Phenotypic and Genome-Wide Analysis of an Antibiotic-Resistant Small Colony Variant (SCV) of Pseudomonas aeruginosa
Source: PLoS One. 2011 Dec 15;6(12):e29276. doi: 10.1371/journal.pone.0029276 (PMC3240657; doi:10.1371/journal.pone.0029276)
Supplement: Table S4 — Down-regulated genes in P. aeruginosa PAO-SCV compared to its clonal wild-type PAO1 during stationary phase. (DOC) [file pone.0029276.s007.doc]

| **Table S4. Down-regulated genes in *P. aeruginosa* PAO-SCV compared to its clonal wild-type PAO1 during stationary phase** | | | | |
| --- | --- | --- | --- | --- |
|  |  |  |  |  |
|  |  |  |  |  |
| **PA number** | **Gene name** | **Fold change** | | **Pruduct name** |
|  |  | **Early SP** | **Late SP** |  |
| Adaptation, protection | | | |  |
| PA3331 |  | 3.5 |  | Cytochrome P450 |
| PA3476 | *rhlI* | 2.3 |  | Autoinducer synthesis protein RhlI |
|  |  |  |  |  |
| Amino acid biosynthesis and metabolism | | | |  |
| PA0872 | *phhA* |  | 15.8 | Phenylalanine-4-hydroxylase |
| PA0899 | *aruB* | 3.0 |  | Succinylarginine dihydrolase |
| PA3152 | *hisH2* | 2.3 |  | Glutamine amidotransferase |
| PA4548 | *yfiT*/*dadA* |  | 2.2 | Probable D-amino acid oxidase |
| PA5302 | *dadX* | 2.8 |  | Catabolic alanine racemase |
|  |  |  |  |  |
| Antibiotic resistance and susceptibility | | | |  |
| PA1129 |  |  | 2.3 | Probable fosfomycin resistance protein |
| PA4208 | *opmD* |  | 2.3 | Probable outer membrane protein precursor |
|  |  |  |  |  |
| Biosynthesis of cofactors, prosthetic groups and carriers | | | | |
| PA0518 | *nirM* | 3.1 |  | Cytochrome c-551 precursor |
| PA0996 | *pqsA* | 8.2 |  | Probable coenzyme A ligase |
| PA0997 | *pqsB* | 12.7 |  | Homologous to beta-keto-acyl-acyl-carrier protein synthase |
| PA0998 | *pqsC* | 14.4 |  | Homologous to beta-keto-acyl-acyl-carrier protein synthase |
| PA0999 | *pqsD* | 8.4 |  | 3-oxoacyl-[acyl-carrier-protein] synthase III |
| PA1000 | *pqsE* | 4.1 |  | Quinolone signal response protein |
| PA1001 | *phnA* | 5.2 |  | Anthranilate synthase component I |
| PA1002 | *phnB* | 5.7 |  | Anthranilate synthase component II |
| PA2587 | *pqsH* | 2.2 |  | Probable FAD-dependent monooxygenase |
|  |  |  |  |  |
| Carbon compound catabolism | | |  |  |
| PA2008 | *fahA* |  | 4.2 | Fumarylacetoacetase |
| PA2009 | *hmgA* |  | 5.8 | Homogentisate 1,2-dioxygenase |
| PA2300 | *chiC* |  | 3.9 | Chitinase |
| PA5131 | *pgm* |  | 2.1 | Phosphoglycerate mutase |
| PA5427 | *adhA* |  | 5.7 | Alcohol dehydrogenase |
|  |  |  |  |  |
| Cell wall / LPS / capsule | |  |  |  |
| PA3145 | *wbpL* | 2.2 |  | Glycosyltransferase WbpL |
| PA3147 | *wbpJ* | 2.5 |  | Probable glycosyl transferase WbpJ |
| PA3148 | *wbpI* | 2.5 |  | Probable UDP-N-acetylglucosamine 2-epimerase WbpI |
| PA3149 | *wbpH* | 3.8 | 2.2 | Probable glycosyltransferase WbpH |
| PA3150 | *wbpG* | 2.3 |  | LPS biosynthesis protein WbpG |
| PA5010 | *waaG* |  | 2.2 | UDP-glucose:(heptosyl) LPS alpha 1,3-glucosyltransferase WaaG |
|  |  |  |  |  |
| Central intermediary metabolism | | |  |  |
| PA0710 | *gloA2* | 17.2 | 3.6 | Lactoylglutathione lyase |
| PA1780 | *nirD* |  | 2.1 | Assimilatory nitrite reductase small subunit |
| PA2195 | *hcnC* | 4.1 |  | Hydrogen cyanide synthase HcnC |
| PA4130 |  |  | 3.5 | Probable sulfite or nitrite reductase |
|  |  |  |  |  |
| Chaperones and heat shock proteins | | |  |  |
| PA5053 | *hslV* |  | 3.6 | Heat shock protein HslV |
|  |  |  |  |  |
| Energy metabolism | |  |  |  |
| PA3394 | *nosF* |  | 2.4 | NosF protein |
| PA4133 | *ccoN*/*fixN*/*cytN* |  | 4.9 | Cytochrome c oxidase subunit (cbb3-type) |
|  |  |  |  |  |
| Fatty acid and phospholipid metabolism | | | |  |
| PA1869 |  | 3.9 |  | Probable acyl carrier protein |
| PA3334 |  | 3.9 | 2.2 | Probable acyl carrier protein |
|  |  |  |  |  |
| Membrane proteins | |  |  |  |
| PA0345 |  |  | 2.1 | Hypothetical protein |
| PA1058 | *phaF* |  | 2.3 | Conserved hypothetical protein |
| PA2331 |  |  | 4.0 | Hypothetical protein |
| PA2662 |  |  | 2.1 | Conserved hypothetical protein |
| PA3278 |  | 4.0 |  | Hypothetical protein |
| PA4067 | *oprG* | 3.3 |  | Outer membrane protein OprG precursor |
| PA4205 | *mexG* |  | 2.5 | Hypothetical protein |
| PA4601 | *morA* |  | 2.1 | Motility regulator |
| PA5113 |  | 2.0 |  | Hypothetical protein |
| PA5114 |  | 2.3 |  | Hypothetical protein |
|  |  |  |  |  |
| Motility and Attachment | |  |  |  |
| PA1087 | *flgL* | 2.2 |  | Flagellar hook-associated protein type 3 FlgL |
|  |  |  |  |  |
| Nucleotide biosynthesis and metabolism | | | |  |
| PA1920 | *nrdD* |  | 2.1 | Class III (anaerobic) ribonucleoside-triphosphate reductase subunit, NrdD |
| PA2962 |  |  | 2.3 | Thymidylate kinase |
|  |  |  |  |  |
| Protein secretion/export apparatus | | |  |  |
| PA3099 | *xcpV* |  | 2.4 | General secretion pathway protein I |
|  |  |  |  |  |
| Putative enzymes | |  |  |  |
| PA0836 | *ackA* |  | 3.6 | Acetate kinase |
| PA1885 |  |  | 2.4 | Conserved hypothetical protein |
| PA2069 |  |  | 2.6 | Probable carbamoyl transferase |
| PA2298 |  |  | 2.0 | Probable oxidoreductase |
| PA2305 |  | 2.0 | 2.3 | Probable non-ribosomal peptide synthetase |
| PA2843 |  |  | 2.0 | Probable aldolase |
| PA3444 | *ssuD* | 2.1 |  | Conserved hypothetical protein |
| PA4089 |  |  | 2.3 | Probable short-chain dehydrogenase |
| PA4131 |  | 19.7 | 3.3 | Probable iron-sulfur protein |
| PA4217 | *phzS* |  | 4.8 | Flavin-containing monooxygenase |
| PA5384 |  |  | 2.4 | Probable lipolytic enzyme |
|  |  |  |  |  |
| Related to phage, transposon, or plasmid | | | |  |
| PA0616 |  | 3.7 | 3.1 | Hypothetical protein |
| PA0617 |  | 3.2 | 2.0 | Probable bacteriophage protein |
| PA0618 |  | 2.3 |  | Probable bacteriophage protein |
| PA0619 |  | 2.8 |  | Probable bacteriophage protein |
| PA0620 |  | 4.1 |  | Probable bacteriophage protein |
| PA0621 |  | 3.7 |  | Conserved hypothetical protein |
| PA0622 |  | 4.2 | 4.1 | Probable bacteriophage protein |
| PA0623 |  | 3.2 |  | Probable bacteriophage protein |
| PA0624 |  | 2.4 |  | Hypothetical protein |
| PA0625 |  | 2.4 | 2.2 | Hypothetical protein |
| PA0627 |  | 2.1 |  | Conserved hypothetical protein |
| PA0628 |  | 2.5 |  | Conserved hypothetical protein |
| PA0630 |  | 2.2 |  | Hypothetical protein |
| PA0631 |  | 2.1 |  | Hypothetical protein |
| PA0633 |  | 4.3 |  | Hypothetical protein |
| PA0635 |  | 2.5 |  | Hypothetical protein |
| PA0636 |  | 3.1 | 2.0 | Hypothetical protein |
| PA0639 |  | 2.1 |  | Hypothetical protein |
| PA0640 |  | 2.1 |  | Conserved hypothetical protein |
| PA0642 |  | 2.1 |  | Hypothetical protein |
| PA0646 |  | 2.8 | 2.7 | Hypothetical protein |
| PA0647 |  | 2.5 |  | Hypothetical protein |
| PA0718 |  | 2.1 | 5.2 | Hypothetical protein of bacteriophage Pf1 |
| PA0719 |  |  | 2.9 | Hypothetical protein of bacteriophage Pf1 |
| PA0721 |  | 2.6 | 2.1 | Hypothetical protein of bacteriophage Pf1 |
| PA0722 |  |  | 2.9 | Hypothetical protein of bacteriophage Pf1 |
| PA0726 |  |  | 2.2 | Hypothetical protein of bacteriophage Pf1 |
|  |  |  |  |  |
| Secreted Factors (toxins, enzymes, alginate) | | | |  |
| PA0985 |  | 3.7 | 2.2 | Pyocin S5 |
| PA1871 | *lasA* |  | 6.6 | LasA protease precursor |
| PA1901 | *phzC2* |  | 5.7 | Phenazine biosynthesis protein PhzC |
| PA1902 | *phzD2* |  | 2.8 | Phenazine biosynthesis protein PhzD |
| PA1903 | *phzE2* |  | 2.7 | Phenazine biosynthesis protein PhzE |
| PA1904 | *phzF2* |  | 2.8 | Probable phenazine biosynthesis protein |
| PA1905 | *phzG2* |  | 4.1 | Probable pyridoxamine 5'-phosphate oxidase |
| PA3479 | *rhlA* | 2.5 |  | Rhamnosyltransferase chain A |
| PA4211 | *phzB1* |  | 4.7 | Probable phenazine biosynthesis protein |
|  |  |  |  |  |
| Transcription, RNA processing and degradation | | | |  |
| PA2840 | *deaD* |  | 2.1 | Probable ATP-dependent RNA helicase |
|  |  |  |  |  |
| Transcriptional regulators | |  |  |  |
| PA0279 | *ydfF* |  | 3.2 | Probable transcriptional regulator |
| PA1098 | *fleS* |  | 2.4 | Two-component sensor |
| PA1196 |  |  | 5.8 | Probable transcriptional regulator |
| PA2588 |  |  | 2.7 | Probable transcriptional regulator |
| PA3845 |  |  | 2.7 | Probable transcriptional regulator |
| PA5116 |  |  | 2.0 | Probable transcriptional regulator |
|  |  |  |  |  |
| Translation, post-translational modification, degradation | | | | |
| PA3326 |  | 4.3 |  | Probable Clp-family ATP-dependent protease |
| PA4482 | *gatC* |  | 3.3 | Glu-tRNA(Gln) amidotransferase subunit C |
|  |  |  |  |  |
| Transport of small molecules | | |  |  |
| PA0162 | *opdC* | 3.0 |  | Histidine porin OpdC |
| PA2327 |  |  | 2.1 | Probable permease of ABC transporter |
| PA2329 |  |  | 2.6 | Probable ATP-binding component of ABC transporter |
| PA3377 | *phnJ* |  | 2.4 | Conserved hypothetical protein |
| PA4206 | *mexH* |  | 2.8 | Probable Resistance-Nodulation-Cell Division (RND) efflux membrane fusion precursor |
| PA5170 | *arcD* | 4.0 |  | Arginine/ornithine antiporter |
|  |  |  |  |  |
| Hypothetical, unclassified, unknown | | |  |  |
| PA0050 |  | 2.8 |  | Hypothetical protein |
| PA0161 |  | 2.8 | 2.1 | Hypothetical protein |
| PA0174 |  |  | 2.2 | Conserved hypothetical protein |
| PA0200 |  | 4.0 | 5.9 | Hypothetical protein |
| PA0201 |  | 2.6 |  | Hypothetical protein |
| PA0404 | *yqgF* |  | 2.7 | Conserved hypothetical protein |
| PA0614 |  | 5.6 | 2.5 | Hypothetical protein |
| PA0615 |  | 2.1 |  | Hypothetical protein |
| PA0709 |  | 15.6 |  | Hypothetical protein |
| PA0711 |  | 2.2 |  | Hypothetical protein |
| PA0713 |  | 18.0 | 2.8 | Hypothetical protein |
| PA0714 |  | 3.9 |  | Hypothetical protein |
| PA0938 |  | 2.3 |  | Hypothetical protein |
| PA1093 | *flaG* | 3.7 |  | Hypothetical protein |
| PA1095 |  | 2.8 |  | Hypothetical protein |
| PA1096 |  | 2.7 |  | Hypothetical protein |
| PA1355 |  |  | 2.8 | Hypothetical protein |
| PA1414 |  | 2.1 |  | Hypothetical protein |
| PA1656 |  | 2.3 |  | Hypothetical protein |
| PA1657 |  | 7.2 |  | Conserved hypothetical protein |
| PA1666 |  | 2.5 |  | Hypothetical protein |
| PA1668 |  | 2.1 |  | Hypothetical protein |
| PA1913 |  | 2.4 |  | Hypothetical protein |
| PA2274 |  |  | 3.7 | Hypothetical protein |
| PA2288 |  |  | 2.2 | Hypothetical protein |
| PA2384 |  |  | 2.8 | Hypothetical protein |
| PA2459 |  | 2.5 |  | Hypothetical protein |
| PA2462 |  | 2.5 |  | Hypothetical protein |
| PA3129 | *yohI* | 2.1 |  | Conserved hypothetical protein |
| PA3177 |  |  | 2.2 | Hypothetical protein |
| PA3273 |  |  | 2.1 | Hypothetical protein |
| PA3329 |  | 2.4 |  | Hypothetical protein |
| PA3332 |  | 2.8 |  | Conserved hypothetical protein |
| PA3413 | *yebG* | 2.2 |  | Conserved hypothetical protein |
| PA3493 | *rnfG* |  | 2.5 | Conserved hypothetical protein |
| PA3501 |  |  | 2.6 | Hypothetical protein |
| PA3613 |  | 2.2 |  | Hypothetical protein |
| PA3662 |  | 3.3 |  | Hypothetical protein |
| PA3719 |  |  | 4.4 | Hypothetical protein |
| PA3722 |  | 2.7 |  | Hypothetical protein |
| PA3880 |  | 4.9 | 2.1 | Conserved hypothetical protein |
| PA3904 |  | 3.4 |  | Hypothetical protein |
| PA3908 |  | 2.3 |  | Hypothetical protein |
| PA3911 | *yhbT* | 2.5 |  | Conserved hypothetical protein |
| PA4129 |  |  | 4.3 | Hypothetical protein |
| PA4132 |  | 7.5 | 2.2 | Conserved hypothetical protein |
| PA4134 |  | 6.3 | 4.3 | Hypothetical protein |
| PA4141 |  | 2.7 | 7.9 | Hypothetical protein |
| PA4220 | *fptB* |  | 4.7 | Hypothetical protein |
| PA4326 |  | 2.2 |  | Hypothetical protein |
| PA4348 |  | 3.4 | 3.7 | Conserved hypothetical protein |
| PA4487 |  | 2.8 |  | Conserved hypothetical protein |
| PA4577 |  | 2.6 | 5.9 | Hypothetical protein |
| PA4773 |  | 2.4 |  | Hypothetical protein |
| PA4782 |  | 2.2 |  | Hypothetical protein |
| PA5228 | *ygfA* |  | 2.5 | Conserved hypothetical protein |
| PA5232 | *yhiI* | 3.0 |  | Conserved hypothetical protein |
| PA5303 |  | 4.0 |  | Conserved hypothetical protein |
| PA5405 |  |  | 2.9 | Hypothetical protein |
| PA5441 |  |  | 2.5 | Hypothetical protein |
| PA5460 |  |  | 2.7 | Hypothetical protein |
| PA5566 |  |  | 2.4 | Hypothetical protein |

*^a^* PA number, gene name and product name are identified through Pseudomonas Genome Database (http://www.pseudomonas.com).

*^b^*  Selected genes with significant expression changes in a magnitude of at least 2-fold are listed (*P* value less than 0.05). SP, stationary phase.

*^c^* LPS, lipopolysaccharide; FAD, flavin adenine dinucleotide.
