# Supplementary material for: Phenotypic and Genome-Wide Analysis of an Antibiotic-Resistant Small Colony Variant (SCV) of Pseudomonas aeruginosa
Source: PLoS One. 2011 Dec 15;6(12):e29276. doi: 10.1371/journal.pone.0029276 (PMC3240657; doi:10.1371/journal.pone.0029276)
Supplement: Table S5 — Differential expression of selected genes in P. aeruginosa PAO-SCV compared to its clonal wild-type PAO1 during stationary phase. (DOC) [file pone.0029276.s008.doc]

| **TableS5. Differential expression of selected genes in *P. aeruginosa* PAO-SCV compared to its clonal wild-type PAO1 during stationary phase** | | | | |
| --- | --- | --- | --- | --- |
|  |  |  |  |  |
| **PA number** | **Gene name** | **Fold change** | | **Pruduct name** |
|  |  | **Early SP** | **Late SP** |  |
| **Antibiotic resistance-related genes** | | | |  |
| PA0424 | *mexR* | 9.2 |  | Multidrug resistance operon repressor MexR |
| PA0425 | *mexA* | 3.3 | 2.2 | RND multidrug efflux protein MexA precursor |
| PA0427 | *oprM* | 2.4 |  | Outer membrane protein OprM precursor |
| PA0706 | *cat* | 2.9 | 4.5 | Chloramphenicol acetyltransferase |
| PA1178 | *oprH* | 4.3 | 5.1 | PhoP/Q and low Mg2+ inducible outer membrane protein H1 precursor |
| PA1179 | *phoP* | 6.8 | 14.3 | Two-component response regulator PhoP |
| PA1180 | *phoQ* | 3.7 | 4.5 | Two-component sensor PhoQ |
| PA2018 | *mexY* | 3.9 |  | RND multidrug efflux transporter |
| PA2019 | *mexX* | 8.7 |  | RND multidrug efflux membrane fusion protein precursor |
| PA2020 | *mexZ* | 3.4 | 2.5 | Probable transcriptional regulator |
| PA4205 | *mexG* |  | -2.5 | Hypothetical protein |
| PA4206 | *mexH* |  | -2.8 | Probable Resistance-Nodulation-Cell Division (RND) efflux membrane fusion precursor |
| PA4208 | *opmD* |  | -2.3 | Probable outer membrane protein precursor |
|  |  |  |  |  |
| **Cell wall / LPS / capsule** | | |  |  |
| PA0705 | *migA* | 6.4 | 3.6 | Alpha-1,6-rhamnosyltransferase MigA |
| PA3145 | *wbpL* | -2.2 |  | Glycosyltransferase WbpL |
| PA3147 | *wbpJ* | -2.5 |  | Probable glycosyl transferase WbpJ |
| PA3148 | *wbpI* | -2.5 |  | Probable UDP-N-acetylglucosamine 2-epimerase WbpI |
| PA3149 | *wbpH* | -3.8 | -2.2 | Probable glycosyltransferase WbpH |
| PA3150 | *wbpG* | -2.3 |  | LPS biosynthesis protein WbpG |
| PA3552 | *arnB* | 13.8 | 24.4 | ArnB |
| PA3553 | *arnC* | 7.6 | 10.8 | ArnC |
| PA3554 | *arnA* |  | 9.8 | ArnA |
| PA3555 | *arnD* | 3.2 |  | ArnD |
| PA3556 | *arnT* | 4.2 | 3.9 | Inner membrane L-Ara4N transferase ArnT |
| PA3557 | *arnE* | 2.5 | 2.7 | ArnE |
| PA3558 | *arnF* | 4.1 | 5.4 | ArnF |
| PA4997 | *msbA* | 2.1 |  | Transport protein MsbA |
| PA5010 | *waaG* |  | -2.2 | UDP-glucose:(heptosyl) LPS alpha 1,3-glucosyltransferase WaaG |
| PA5276 | *lppL* |  | 3.0 | Lipopeptide LppL precursor |
| PA5450 | *wzt* |  | 2.0 | ABC subunit of A-band LPS efflux transporter |
|  |  |  |  |  |
| **Transport of small molecules** | | |  |  |
| PA0300 | *spuD* |  | 3.5 | Polyamine transport protein PotF2 |
| PA0302 | *spuF* |  | 2.1 | Polyamine transport protein PotG |
| PA0688 |  |  | 2.4 | Probable binding protein component of ABC transporter |
| PA0693 | *exbB2* |  | 3.0 | Transport protein ExbB2 |
| PA0694 | *exbD2* |  | 2.1 | Transport protein ExbD |
| PA0703 |  |  | 2.2 | Probable major facilitator superfamily (MFS) transporter |
| PA0866 | *aroP2* | 4.6 |  | Aromatic amino acid transport protein AroP2 |
| PA0958 | *oprD* |  | 5.0 | Basic amino acid, basic peptide and imipenem outer membrane porin OprD precursor |
| PA0972 | *tolB* | 2.2 |  | TolB protein |
| PA1070 | *braG* |  | 2.5 | Branched-chain amino acid transport protein BraG |
| PA1074 | *braC* |  | 3.5 | Branched-chain amino acid transport protein BraC |
| PA1342 |  |  | 3.0 | Probable binding protein component of ABC transporter |
| PA1493 | *cysP* |  | 2.5 | Sulfate-binding protein of ABC transporter |
| PA1946 | *rbsB* |  | 8.6 | Binding protein component precursor of ABC ribose transporter |
| PA1947 | *rbsA* |  | 4.2 | Ribose transport protein RbsA |
| PA1948 | *rbsC* |  | 2.2 | Membrane protein component of ABC ribose transporter |
| PA2041 |  | 2.8 |  | Probable amino acid permease |
| PA2760 | *oprQ* |  | 6.1 | Probable outer membrane protein precursor |
| PA3038 | *opdQ* |  | 7.1 | Probable porin |
| PA3186 | *oprB* |  | 6.4 | Glucose/carbohydrate outer membrane porin OprB precursor |
| PA3187 | *gltK* |  | 3.0 | Probable ATP-binding component of ABC transporter |
| PA3188 | *gltG* |  | 3.2 | Probable permease of ABC sugar transporter |
| PA3190 | *gltB* |  | 4.4 | Probable binding protein component of ABC sugar transporter |
| PA3234 | *yjcG* | 5.6 | 2.5 | Probable sodium:solute symporter |
| PA3236 |  |  | 10.6 | Probable glycine betaine-binding protein precursor |
| PA3889 |  | 2.1 |  | Probable binding protein component of ABC transporter |
| PA4023 | *eutP* | 2.3 |  | Probable transport protein |
| PA4456 | *yrbF* | 2.3 |  | Probable ATP-binding component of ABC transporter |
| PA4461 | *yhbG* |  | 2.3 | Probable ATP-binding component of ABC transporter |
| PA4496 |  |  | 3.3 | Probable binding protein component of ABC transporter |
| PA4500 |  |  | 10.6 | Probable binding protein component of ABC transporter |
| PA4502 |  |  | 3.1 | Probable binding protein component of ABC transporter |
| PA4913 |  |  | 2.2 | Probable binding protein component of ABC transporter |
| PA5152 |  |  | 2.9 | Probable ATP-binding component of ABC transporter |
| PA5153 |  |  | 8.0 | Probable periplasmic binding protein |
| PA5167 |  |  | 4.2 | Probable c4-dicarboxylate-binding protein |
| PA5376 |  |  | 2.6 | Probable ATP-binding component of ABC transporter |
|  |  |  |  |  |
| **Protein secretion/export apparatus** | | | |  |
| PA0677 | *hxcW* |  | 2.6 | HxcW putative pseudopilin |
| PA0680 | *hxcV* |  | 2.4 | HxcV putative pseudopilin |
| PA0683 | *hxcY* |  | 2.7 | Probable type II secretion system protein |
| PA0685 | *hxcQ* |  | 4.7 | Probable type II secretion system protein |
| PA0686 | *hxcR* |  | 2.2 | Probable type II secretion system protein |
| PA3099 | *xcpV* |  | -2.4 | General secretion pathway protein I |
| PA4403 | *secA* |  | 3.9 | Secretion protein SecA |
| PA4747 | *secG* |  | 2.0 | Secretion protein SecG |
| PA5128 | *secB* |  | 2.4 | Secretion protein SecB |
|  |  |  |  |  |
| **Motility and Attachment** | | |  |  |
| PA1087 | *flgL* | -2.2 |  | Flagellar hook-associated protein type 3 FlgL |
| PA1093 | *flaG* | -3.7 |  | Hypothetical protein |
| PA1095 |  | -2.8 |  | Hypothetical protein |
| PA1096 |  | -2.7 |  | Hypothetical protein |
| PA1098 | *fleS* |  | -2.4 | Two-component sensor |
| PA4601 | *morA* |  | -2.1 | Motility regulator |
|  |  |  |  |  |
| **Virulence factors** | |  |  |  |
| PA0996 | *pqsA* | -8.2 |  | Probable coenzyme A ligase |
| PA0997 | *pqsB* | -12.7 |  | Homologous to beta-keto-acyl-acyl-carrier protein synthase |
| PA0998 | *pqsC* | -14.4 |  | Homologous to beta-keto-acyl-acyl-carrier protein synthase |
| PA0999 | *pqsD* | -8.4 |  | 3-oxoacyl-[acyl-carrier-protein] synthase III |
| PA1000 | *pqsE* | -4.1 |  | Quinolone signal response protein |
| PA1001 | *phnA* | -5.2 |  | Anthranilate synthase component I |
| PA1002 | *phnB* | -5.7 |  | Anthranilate synthase component II |
| PA1871 | *lasA* |  | -6.6 | LasA protease precursor |
| PA1901 | *phzC2* |  | -5.7 | Phenazine biosynthesis protein PhzC |
| PA1902 | *phzD2* |  | -2.8 | Phenazine biosynthesis protein PhzD |
| PA1903 | *phzE2* |  | -2.7 | Phenazine biosynthesis protein PhzE |
| PA1904 | *phzF2* |  | -2.8 | Probable phenazine biosynthesis protein |
| PA1905 | *phzG2* |  | -4.1 | Probable pyridoxamine 5'-phosphate oxidase |
| PA2195 | *hcnC* | -4.1 |  | Hydrogen cyanide synthase HcnC |
| PA2587 | *pqsH* | -2.2 |  | Probable FAD-dependent monooxygenase |
| PA3479 | *rhlA* | -2.5 |  | Rhamnosyltransferase chain A |
| PA4211 | *phzB1* |  | -4.7 | Probable phenazine biosynthesis protein |
| PA4217 | *phzS* |  | -4.8 | Flavin-containing monooxygenase |
|  |  |  |  |  |
| **Secreted Factors (toxins, enzymes, alginate)** | | | | |
| PA2862 | *lipA* |  | 4.9 | Lactonizing lipase precursor |
| PA4221 | *fptA* | 2.7 |  | Fe(III)-pyochelin outer membrane receptor precursor |
| PA4226 | *pchE* | 2.8 |  | Dihydroaeruginoic acid synthetase PchE |
| PA4228 | *pchD* | 3.8 |  | Pyochelin biosynthesis protein PchD |
| PA4229 | *pchC* | 2.5 |  | Pyochelin biosynthetic protein PchC |
| PA4230 | *pchB* | 3.7 |  | Salicylate biosynthesis protein PchB |
| PA5112 | *estA* |  | 2.4 | Esterase EstA |
|  |  |  |  |  |
| **Transcriptional regulators** | | |  |  |
| PA0167 |  | 2.2 |  | Probable transcriptional regulator |
| PA0676 |  |  | 3.3 | Probable transmembrane sensor |
| PA0701 |  |  | 2.0 | Probable transcriptional regulator |
| PA0707 | *toxR* |  | 2.5 | Transcriptional regulator ToxR |
| PA0762 | *algU* | 2.3 |  | Sigma factor AlgU |
| PA0763 | *mucA* | 2.0 |  | Anti-sigma factor MucA |
| PA0780 | *pruR* | 2.2 |  | Proline utilization regulator |
| PA0797 |  | 2.5 |  | Probable transcriptional regulator |
| PA0876 |  | 2.0 |  | Probable transcriptional regulator |
| PA0929 | *pirR* | 2.2 |  | Two-component response regulator |
| PA1159 |  |  | 4.8 | Probable cold-shock protein |
| PA1504 |  | 2.5 |  | Probable transcriptional regulator |
| PA1754 | *cysB* |  | 3.4 | Transcriptional regulator CysB |
| PA1949 | *rbsR* |  | 2.0 | Ribose operon repressor RbsR |
| PA1978 | *agmR* |  | 6.9 | Probable transcriptional regulator |
| PA2388 | *fpvR* | 2.4 |  | Probable transmembrane sensor |
| PA2622 | *cspD* |  | 3.8 | Cold-shock protein CspD |
| PA2896 |  | 2.2 |  | Probable sigma-70 factor, ECF subfamily |
| PA3161 | *himD* |  | 2.3 | Integration host factor beta subunit |
| PA3385 | *amrZ* | 2.1 |  | Alginate and motility regulator Z |
| PA3476 | *rhlI* | -2.3 | 2.1 | Autoinducer synthesis protein RhlI |
| PA3622 | *rpoS* |  | 4.0 | Sigma factor RpoS |
| PA4723 | *dksA* |  | 2.3 | Suppressor protein DksA |
| PA4764 | *fur* |  | 2.7 | Ferric uptake regulation protein |
| PA4944 | *hfq* |  | 2.8 | Sm-like RNA-binding protein Hfq |
| PA5253 | *algP* |  | 3.2 | Alginate regulatory protein AlgP |
| PA5255 | *algQ* |  | 2.2 | Alginate regulatory protein AlgQ |
| PA5301 | *ycjC* | 2.5 | 2.7 | Probable transcriptional regulator |
| PA5337 | *rpoZ* | 2.1 |  | RNA polymerase omega subunit |
| PA5360 | *phoB* |  | 2.2 | Two-component response regulator PhoB |
| PA5380 | *gbdR* |  | 3.0 | AraC family transcription factor GbdR |
| PA5484 | *kinB* | 2.1 |  | Probable two-component sensor |
|  |  |  |  |  |
| **Translation, post-translational modification, degradation** | | | | |
| PA0019 | *def* |  | 2.1 | Polypeptide deformylase |
| PA1122 | *fms*/*pdf*/*def* | 2.1 | 2.5 | Probable peptide deformylase |
| PA2071 | *fusA2* | 2.1 |  | Elongation factor G |
| PA2612 | *serS* | 2.4 |  | Seryl-tRNA synthetase |
| PA2619 | *infA* | 2.5 | 3.0 | Initiation factor |
| PA2620 | *clpA* | 2.4 |  | ATP-binding protease component ClpA |
| PA2739 | *pheT* | 2.3 |  | Phenylalanyl-tRNA synthetase, beta subunit |
| PA2740 | *pheS* | 2.9 |  | Phenylalanyl-tRNA synthetase, alpha-subunit |
| PA2742 | *rpmI* |  | 4.4 | 50S ribosomal protein L35 |
| PA2755 | *eco* |  | 2.3 | Ecotin precursor |
| PA2851 | *efp* |  | 3.5 | Translation elongation factor P |
| PA3049 | *rmf* |  | 2.7 | Ribosome modulation factor |
| PA3162 | *rpsA* |  | 2.5 | 30S ribosomal protein S1 |
| PA3834 | *valS* |  | 2.1 | Valyl-tRNA synthetase |
| PA3987 | *leuS* |  | 2.7 | Leucyl-tRNA synthetase |
| PA4138 | *tyrS* | 3.1 |  | Tyrosyl-tRNA synthetase |
| PA4242 | *rpmJ* |  | 8.6 | 50S ribosomal protein L36 |
| PA4245 | *rpmD* |  | 2.7 | 50S ribosomal protein L30 |
| PA4246 | *rpsE* |  | 3.3 | 30S ribosomal protein S5 |
| PA4247 | *rplR* |  | 3.4 | 50S ribosomal protein L18 |
| PA4248 | *rplF* |  | 2.1 | 50S ribosomal protein L6 |
| PA4249 | *rpsH* |  | 2.0 | 30S ribosomal protein S8 |
| PA4252 | *rplX* |  | 3.8 | 50S ribosomal protein L24 |
| PA4253 | *rplN* |  | 2.5 | 50S ribosomal protein L14 |
| PA4254 | *rpsQ* |  | 3.2 | 30S ribosomal protein S17 |
| PA4262 | *rplD* |  | 2.9 | 50S ribosomal protein L4 |
| PA4264 | *rpsJ* |  | 3.2 | 30S ribosomal protein S10 |
| PA4265 | *tufA* |  | 2.7 | Elongation factor Tu |
| PA4268 | *rpsL* |  | 2.8 | 30S ribosomal protein S12 |
| PA4274 | *rplK* |  | 2.6 | 50S ribosomal protein L11 |
| PA4433 | *rplM* |  | 6.7 | 50S ribosomal protein L13 |
| PA4563 | *rpsT* |  | 3.0 | 30S ribosomal protein S20 |
| PA4567 | *rpmA* |  | 2.0 | 50S ribosomal protein L27 |
| PA4568 | *rplU* |  | 4.2 | 50S ribosomal protein L21 |
| PA4671 | *rplY* |  | 2.0 | Probable ribosomal protein L25 |
| PA4935 | *rpsF* |  | 2.7 | 30S ribosomal protein S6 |
| PA5049 | *rpmE* |  | 3.0 | 50S ribosomal protein L31 |
| PA5316 | *rpmB* |  | 3.4 | 50S ribosomal protein L28 |
|  |  |  |  |  |
| **Related to phage, transposon, or plasmid** | | | |  |
| PA0616 |  | -3.7 | -3.1 | Hypothetical protein |
| PA0617 |  | -3.2 | -2.0 | Probable bacteriophage protein |
| PA0618 |  | -2.3 |  | Probable bacteriophage protein |
| PA0619 |  | -2.8 |  | Probable bacteriophage protein |
| PA0620 |  | -4.1 |  | Probable bacteriophage protein |
| PA0621 |  | -3.7 |  | Conserved hypothetical protein |
| PA0622 |  | -4.2 | -4.1 | Probable bacteriophage protein |
| PA0623 |  | -3.2 |  | Probable bacteriophage protein |
| PA0624 |  | -2.4 |  | Hypothetical protein |
| PA0625 |  | -2.4 | -2.2 | Hypothetical protein |
| PA0627 |  | -2.1 |  | Conserved hypothetical protein |
| PA0628 |  | -2.5 |  | Conserved hypothetical protein |
| PA0630 |  | -2.2 |  | Hypothetical protein |
| PA0631 |  | -2.1 |  | Hypothetical protein |
| PA0633 |  | -4.3 |  | Hypothetical protein |
| PA0635 |  | -2.5 |  | Hypothetical protein |
| PA0636 |  | -3.1 | -2.0 | Hypothetical protein |
| PA0639 |  | -2.1 |  | Hypothetical protein |
| PA0640 |  | -2.1 |  | Conserved hypothetical protein |
| PA0642 |  | -2.1 |  | Hypothetical protein |
| PA0646 |  | -2.8 | -2.7 | Hypothetical protein |
| PA0647 |  | -2.5 |  | Hypothetical protein |
| PA0718 |  | -2.1 | -5.2 | Hypothetical protein of bacteriophage Pf1 |
| PA0719 |  |  | -2.9 | Hypothetical protein of bacteriophage Pf1 |
| PA0721 |  | -2.6 | -2.1 | Hypothetical protein of bacteriophage Pf1 |
| PA0722 |  |  | -2.9 | Hypothetical protein of bacteriophage Pf1 |
| PA0726 |  |  | -2.2 | Hypothetical protein of bacteriophage Pf1 |

*^a^* PA number, gene name and product name are identified through Pseudomonas Genome Database (http://www.pseudomonas.com).

*^b^*  Selected genes with significant expression changes in a magnitude of at least 2-fold are listed (*P* value less than 0.05). Up-regulated genes are shown as positives numbers; Down-regulated genes are given as negative numbers. SP, stationary phase.

*^c^* RND, resistance-nodulation-cell division; LPS, lipopolysaccharide; ABC, ATP-binding cassette; MFS, major facilitator superfamily; FAD, flavin adenine dinucleotide; ECF, extracytoplamic function.
